# ADMETlab 2.0: an integrated online platform for accurate and comprehensive predictions of ADMET properties

**DOI:** 10.1093/nar/gkab255

**Published:** 2021-04-24

**Authors:** Guoli Xiong, Zhenxing Wu, Jiacai Yi, Li Fu, Zhijiang Yang, Changyu Hsieh, Mingzhu Yin, Xiangxiang Zeng, Chengkun Wu, Aiping Lu, Xiang Chen, Tingjun Hou, Dongsheng Cao

**Affiliations:** Xiangya School of Pharmaceutical Sciences, Central South University, Changsha 410013, Hunan, China; Hangzhou Institute of Innovative Medicine, College of Pharmaceutical Sciences, Zhejiang University, Hangzhou 310058, Zhejiang, China; College of Computer, National University of Defense Technology, Changsha 410073, Hunan, China; Xiangya School of Pharmaceutical Sciences, Central South University, Changsha 410013, Hunan, China; Xiangya School of Pharmaceutical Sciences, Central South University, Changsha 410013, Hunan, China; Tencent Quantum Laboratory, Tencent, Shenzhen 518057, Guangdong, China; Department of Dermatology, Hunan Engineering Research Center of Skin Health and Disease, Hunan Key Laboratory of Skin Cancer and Psoriasis, Xiangya Hospital, Central South University, Changsha 410008, Hunan, China; Deparment of Computer Science, Hunan University, Changsha 410082, Hunan, China; College of Computer, National University of Defense Technology, Changsha 410073, Hunan, China; Institute for Advancing Translational Medicine in Bone and Joint Diseases, School of Chinese Medicine, Hong Kong Baptist University, Hong Kong SAR, China; Department of Dermatology, Hunan Engineering Research Center of Skin Health and Disease, Hunan Key Laboratory of Skin Cancer and Psoriasis, Xiangya Hospital, Central South University, Changsha 410008, Hunan, China; Hangzhou Institute of Innovative Medicine, College of Pharmaceutical Sciences, Zhejiang University, Hangzhou 310058, Zhejiang, China; Xiangya School of Pharmaceutical Sciences, Central South University, Changsha 410013, Hunan, China; Institute for Advancing Translational Medicine in Bone and Joint Diseases, School of Chinese Medicine, Hong Kong Baptist University, Hong Kong SAR, China

## Abstract

Because undesirable pharmacokinetics and toxicity of candidate compounds are the main reasons for the failure of drug development, it has been widely recognized that absorption, distribution, metabolism, excretion and toxicity (ADMET) should be evaluated as early as possible. In silico ADMET evaluation models have been developed as an additional tool to assist medicinal chemists in the design and optimization of leads. Here, we announced the release of ADMETlab 2.0, a completely redesigned version of the widely used AMDETlab web server for the predictions of pharmacokinetics and toxicity properties of chemicals, of which the supported ADMET-related endpoints are approximately twice the number of the endpoints in the previous version, including 17 physicochemical properties, 13 medicinal chemistry properties, 23 ADME properties, 27 toxicity endpoints and 8 toxicophore rules (751 substructures). A multi-task graph attention framework was employed to develop the robust and accurate models in ADMETlab 2.0. The batch computation module was provided in response to numerous requests from users, and the representation of the results was further optimized. The ADMETlab 2.0 server is freely available, without registration, at https://admetmesh.scbdd.com/.

## Graphical Abstract

**Graphical Abstract:**
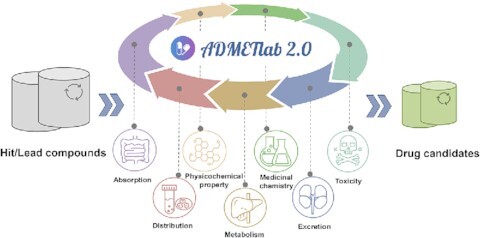
ADMETlab 2.0 assists medicinal chemists in the design and optimization of lead compounds.

## INTRODUCTION

A successful drug should achieve a finely tuned combination of biochemical behavior, pharmacokinetics and safety. In addition to high potency and selectivity, desirable absorption, distribution, metabolism, excretion and toxicity (ADMET) profile is equally critical to the success of a drug candidate (1–3). More specifically, an ideal drug should be taken appropriately into the body, distributed reasonably to various tissues and organs, metabolized in a way that does not immediately remove its activity, eliminated in a suitable manner, and confirmed non-toxicity (4). These issues seem distinct but closely interrelated, covering the whole process from administration to elimination.

Traditionally, the measurement of the ADMET properties for a drug candidate was usually scheduled after its potency towards a specific target was determined (5). Unfortunately, undesirable adverse effects were often detected in this stage, and therefore a new round of molecular design and syntheses need to be conducted or even the project has to be terminated thoroughly. It was estimated that the contribution of ADMET deficiencies to attrition in drug development reached up to 50% in the 1990s (6,7), which reminded us of the importance of ADMET evaluation in the process from chemicals to drugs. Currently, the potency and ADMET profiles of molecules are usually tested at the same stage, and thus undesirable compounds can be excluded at an earlier stage of drug discovery and development (8,9). However, the testing capacity of *in vitro* and *in vivo* ADMET assays seems stretched thin when confronted with the sheer volume of biological screening data, although extensive efforts have been dedicated to ramping up the testing capacity (10). Additionally, the time and cost for experimental assays is another burden, especially for drug discovery pipelines with limited resources.

With the continuous accumulation of experimental ADMET data, a high number of *in silico* prediction models for many endpoints have been developed to assist ADMET evaluation efficiently. More concretely, they could assist medicinal chemists from two aspects: 1) excluding undesirable compounds at the drug design stage; 2) acquiring timely feedback of ADMET information for lead optimization. The past several years have witnessed many *in silico* studies concerning ADMET parameters. Meanwhile, a variety of web tools have been developed and used in drug discovery applications, such as ADMETlab (11), FAF-Drugs4 (12), admetSAR (13), SwissADME (14), ProTox-II (15), pkCSM (16), etc.

Thereinto, the webserver ADMETlab was released by our team in 2018. Armed with high-quality experimental data and tailored quantitative structure-property relationship (QSPR) models, it allows users to perform multiple drug-likeness analyses and the predictions of most ADMET-related properties. During the past three years, it has been widely used for ADMET assessment, serving >50 000 users around the world, with millions of entries computed. However, some shortcomings of ADMETlab still need to be improved, such as redundant modules, incomplete endpoints, unclear representation of results, and so on. As part of our continual efforts to provide the community with a comprehensive, accurate and efficient online platform for the evaluation of ADMET-related parameters for chemicals, we updated the server to version 2.0, overcoming all known shortcomings of the old version while maintaining its battle-tested advantages. ADMETlab 2.0 currently supports two computational modes: single-molecule evaluation and batch screening, allowing for the calculation of 88 ADMET-related parameters, including 17 physicochemical properties, 13 medicinal chemistry properties, 23 ADME properties, 27 toxicity endpoints and 8 toxicophore rules (751 substructures). It is backed up by the robust QSPR models trained by the multi-task graph attention (MGA) framework based on high-quality experimental ADMET data. To sum up, the upgraded version is believed to have greater capacity to assist medicinal chemists in accelerating the drug research and development (R&D) process. ADMETlab 2.0 is implemented as a publicly available web server with an intuitive interface and can be freely accessed at https://admetmesh.scbdd.com/.

## PROGRAM DESCRIPTION AND METHODS

### Software implementation

ADMETlab 2.0 was built using the Python web framework of Django and deployed on an elastic compute service from Aliyun running an Ubuntu Linux system. The web access was enabled via the Nginx web server and the interactions between Django and proxy server were supported by uwsgi. This application was developed based on the Model-View-Template (MVT) framework. The model layer maps the business objects to the database objects. The view layer is a business logic layer, responsible for performing the access to the deep learning models, delivering the data to be shown on the template layer, and handling the upload and download of files. The template layer provides the visualization of results, page rendering, integration of documentation, etc. The uploaded and downloaded files, pre-trained models and model predictions were stored in the server. The prediction models were built with the Python programming language. The deep learning packages, PyTorch and DGL, were used in model implementation. Additionally, the RDKit package was employed to provide various cheminformatics support. The server has been successfully tested on the recent version of Mozilla Firefox, Google Chrome and Apple Safari.

### Input and output

ADMETlab 2.0 provides a convenient and easy-to-use interface for users. Two services, *Evaluation* and *Screening*, are designed to support single-molecule and batch evaluation, whose input parameters and output information will be elaborated respectively.

In the *Evaluation* pattern, two molecular submission approaches are provided by pasting the SMILES string or drawing the chemical structure with the help of JMSE molecule editor (17). Once a user submits the job, the webserver will automatically standardize the input SMILES strings and compute all the endpoints. The prediction results are mainly displayed in the tabular format in the browser, with the 2D molecular structure and a radar plot summarizing the physicochemical quality of the compound. For those endpoints predicted by the regression models, such as Caco-2 permeability, plasma protein binding, etc., concrete predictive values are provided. For the endpoints predicted by the classification models, such as Pgp-inhibitor, hERG Blocker, etc., the prediction probability values are transformed into six symbols: 0-0.1(−−−), 0.1-0.3(−−), 0.3-0.5(−), 0.5-0.7(+), 0.7-0.9(++), and 0.9-1.0(+++). Usually, the token ‘+++’ or ‘++’ represents the molecule is more likely to be toxic or defective, while ‘−−−’ or ‘−−’ represents nontoxic or appropriate. Here, we do not recommend trusting predictions symbolled by ‘+’ or ‘−' (probably values in 0.3-0.7), and corresponding molecules require further assessment. The substructural rules available in the webserver, such as PAINS, SureChEMBL Rule, etc., were implemented using the SMARTS recognition capability of RDKit function. And the calculation of physicochemical and medicinal chemistry endpoints was based on the python library Scopy (18), following the parameters reported in corresponding original papers strictly. If the number of alerts is not zero, users can click the *DETAIL* button to check the undesirable substructures in the molecule. Finally, the full result file can be downloaded from the website in CSV or PDF format.

In the *Screening* pattern, two molecular submission approaches are provided by entering a list of SMILES strings or uploading a SDF or TXT formatted file. It should be noted that the file should only contain molecules without column headers and molecular indexes, otherwise the server may declare invalid input type. After all the predictions are completed, the results for each input molecule will be presented on a separate row, containing the assigned index, SMILES string, 2D molecular structure, and a *View* button. The prediction details can be accessed by clicking the *View* button of the corresponding molecule that links to the single-molecule evaluation page. These results can also be downloaded as a CSV-formatted file to the user's computer, where concrete probably values of classification endpoints are provided to enable the users to define their own thresholds to filter out deficient compounds with different levels of reliability. A typical ADMETlab 2.0 task for 1000 molecules requires ∼84 s, but it may also depend on the complexity of molecules.

### Data collection

To obtain as much data as possible for model training, we conducted a comprehensive data retrieval by using different ADMET-related keywords. The data sources included open-access bioactivity databases, such as ChEMBL (19), PubChem (20) and OCHEM (21), peer-reviewed literature, and freely accessible software Toxicity Estimation Software Tools (TEST) developed by the U.S. Environmental Protection Agency (22). In data curation, we filtered off organometallic compounds, isomeric mixtures and chemical mixtures, neutralized salts, eliminated counterions and transformed SMILES strings into canonical form. Subsequently, the molecules with more than 128 atoms (unsuitable for GNN model training) and duplicated entries were removed, leaving a high-quality dataset collection of 0.25M entries spanning 53 ADMET-related endpoints. The scaffold analysis indicated a high level of the structural diversity of the training sets, and the models developed with such datasets may have good prediction coverage for structurally diverse compounds. More details of the modeling data and scaffold analysis are provided in Supplementary Tables S1 and S2.

### Model validation

In this update, a total of 53 prediction models were implemented, including 40 classification models and 13 regression models. For each endpoint, the dataset was split into the training, validation and test sets by a ratio of 8:1:1, and stratified sampling was used when partitioning the data for classification to keep the ratio of the positive and negative instances in the three subsets balanced. The larger part was used for training, and the validation and test sets were used to optimize the hyperparameters and test the predictive capacity of each model, respectively. As for the evaluation parameters, we selected accuracy (ACC), specificity (SP), sensitivity (SE), the area under the ROC curve (AUC), and the Matthews correlation coefficient (MCC) (23) for the classification models, and *R*-square (*R*^2^), mean absolute error (MAE), and root mean squared error (RMSE) for the regression models. To obtain robust and accurate prediction models, the model training process was repeated ten times with random data splitting. The best performing models were incorporated into the online platform, and different performance measures for the classification and regression models were separately summarized in Tables 1 and 2, respectively. For the classification models, most of them achieved an AUC of 0.85 or higher, except for some cytochrome enzyme endpoints such as CYP1A2 substrate and CYP2C9 substrate. Meanwhile, these models yielded satisfactory prediction accuracy, with 27 models reaching the ACC values above 0.8. The average MCC value of these models is 0.53 and 50% of models obtain a MCC of more than 0.5. The specificity and sensitivity of most models were relatively balanced, except for some endpoints in the Toxicology in the 21st Century (Tox21) dataset, which was probably due to the imbalanced labeled data. For the regression models, most of them achieved an *R*^2^ above 0.72. Nevertheless, some endpoints, such as Clearance and LC_50_DM, had relatively limited historical data for model training, rendering the underperformance of the corresponding models. The complete performance summary for the training and test sets are listed in Supplementary Tables S3 and S4. Generally, these models can give relatively accurate predictions for the ADMET-related properties of chemicals.

**Table 1.** Performance of the classification models incorporated into the ADMETlab 2.0 platform

Category	Model	AUC	ACC	MCC	Specificity	Sensitivity
**Absorption**	Pgp-inhibitor	0.922	0.867	0.723	0.844	0.882
	Pgp-substrate	0.840	0.768	0.538	0.705	0.828
	HIA	0.866	0.924	0.687	0.800	0.942
	F_20%_	0.833	0.750	0.414	0.680	0.773
	F_30%_	0.848	0.802	0.580	0.794	0.806
**Distribution**	BBB Penetration	0.908	0.862	0.718	0.824	0.891
**Metabolism**	CYP1A2 inhibitor	0.928	0.852	0.704	0.848	0.857
	CYP1A2 substrate	0.737	0.649	0.298	0.632	0.667
	CYP2C19 inhibitor	0.913	0.839	0.679	0.813	0.869
	CYP2C19 substrate	0.758	0.654	0.300	0.667	0.636
	CYP2C9 inhibitor	0.919	0.841	0.671	0.823	0.878
	CYP2C9 substrate	0.725	0.707	0.386	0.776	0.606
	CYP2D6 inhibitor	0.892	0.824	0.558	0.823	0.828
	CYP2D6 substrate	0.847	0.775	0.553	0.733	0.818
	CYP3A4 inhibitor	0.921	0.832	0.659	0.825	0.841
	CYP3A4 substrate	0.776	0.713	0.437	0.820	0.608
**Excretion**	T_1/2_	0.801	0.727	0.478	0.658	0.827
**Toxicity**	hERG Blockers	0.943	0.889	0.778	0.869	0.909
	H-HT	0.814	0.720	0.461	0.814	0.650
	DILI	0.924	0.894	0.793	0.826	0.958
	AMES Toxicity	0.902	0.807	0.606	0.732	0.865
	Rat Oral Acute Toxicity Toxicity	0.853	0.778	0.549	0.769	0.793
	FDAMDD	0.804	0.736	0.471	0.734	0.737
	Skin Sensitization	0.707	0.775	0.462	0.539	0.889
	Carcinogencity	0.788	0.731	0.476	0.623	0.843
	Eye Corrosion	0.983	0.957	0.908	0.965	0.944
	Eye Irritation	0.982	0.952	0.876	0.918	0.964
	Respiratory Toxicity	0.828	0.764	0.514	0.732	0.786
	NR-AR	0.886	0.890	0.348	0.896	0.731
	NR-AR-LBD	0.915	0.936	0.472	0.942	0.783
	NR-AhR	0.943	0.862	0.573	0.858	0.896
	NR-Aromatase	0.852	0.849	0.264	0.859	0.615
	NR-ER	0.771	0.815	0.320	0.845	0.567
	NR-ER-LBD	0.850	0.903	0.364	0.918	0.618
	NR-PPAR-gamma	0.893	0.896	0.344	0.901	0.750
	SR-ARE	0.863	0.827	0.469	0.850	0.701
	SR-ATAD5	0.874	0.919	0.361	0.929	0.640
	SR-HSE	0.907	0.868	0.393	0.875	0.750
	SR-MMP	0.927	0.897	0.660	0.908	0.835
	SR-p53	0.881	0.841	0.365	0.849	0.723

**Table 2.** Performance of the regression models incorporated into the ADMETlab 2.0 platform

Category	Model	*R* ^2^	RMSE	MAE
**Physicochemical property**	Log *S*	0.854	0.850	0.588
	Log *D*7.4	0.892	0.462	0.347
	Log *P*	0.957	0.357	0.256
**Absorption**	Caco-2 permeability	0.746	0.307	0.222
	MDCK permeability	0.731	0.291	0.199
**Distribution**	PPB	0.733	0.135	0.834
	VD	0.782	0.670	0.457
	Fu	0.763	0.367	0.263
**Excretion**	CL	0.678	3.375	2.240
**Toxicity**	Bioconcentration factor	0.786	0.603	0.435
	IGC_50_	0.723	0.496	0.335
	LC_50_FM	0.745	0.863	0.643
	LC_50_DM	0.524	0.994	0.692

Besides, we also implemented more rigorous leave-cluster-out validation, where the molecules were grouped into clusters according to the Murcko scaffolds. The scaffolds of validation and test sets were excluded from the training set. The classification endpoints could obtain an average MCC of 0.47, and regression endpoints could obtain an average *R*^2^ of 0.65, suggesting that the knowledge learned from the training set has the potential to be transferred to other different chemical structures and the predictive ability of our webserver is reliable. More detailed results of leave-cluster-out validation are listed in Supplementary Tables S5 and S6.

### Multi-task graph attention (MGA) framework

Traditional multitask graph neural network (GNN) methods usually handle homogeneous tasks, such as pure regression or classification tasks. However, in ADMET prediction, both regression tasks and classification tasks are needed. Therefore, a multi-task graph attention (MGA) framework was used to simultaneously learn the regression and classification tasks for ADMET predictions in this study. An overview of the Multi-task Graph Attention framework is shown in Supplementary Figure S1. MGA is composed of the input, relation graph convolution network (RGCN) layers (24), attention layer, and fully-connected (FC) layers. In the input, a node represents the information of an atom, and after passing the RGCN layers, the node represents the general features of the circular substructure centered on the atom. The attention layers can assign different attention weights to different substructures, and then generate the customized fingerprints from the general features for a specific task. It's worth noting that MGA has different attention layers for different tasks and can generate customized fingerprints for different tasks. The FC layers predict the corresponding tasks based on the customized fingerprints. The classification and regression tasks adopt different loss functions (*loss_c* and *loss_r*), and the loss function of MGA is a combination of *loss_c* and *loss_r* (25). To alleviate data-imbalance problem, we employed BCEWithLogitsLoss loss function and attached corresponding weights of positive examples (pos_weight) according to the proportion of positive and negative samples. We used the empirical hyperparameters of MGA to construct the ADMET prediction models and more information on the MGA method can be seen in Supplementary Data. The implementation and empirical hyperparameters of MGA can be found at https://github.com/wzxxxx/MGA.

## NEW DEVELOPMENTS IN ADMETlab 2.0

**Figure 1.**
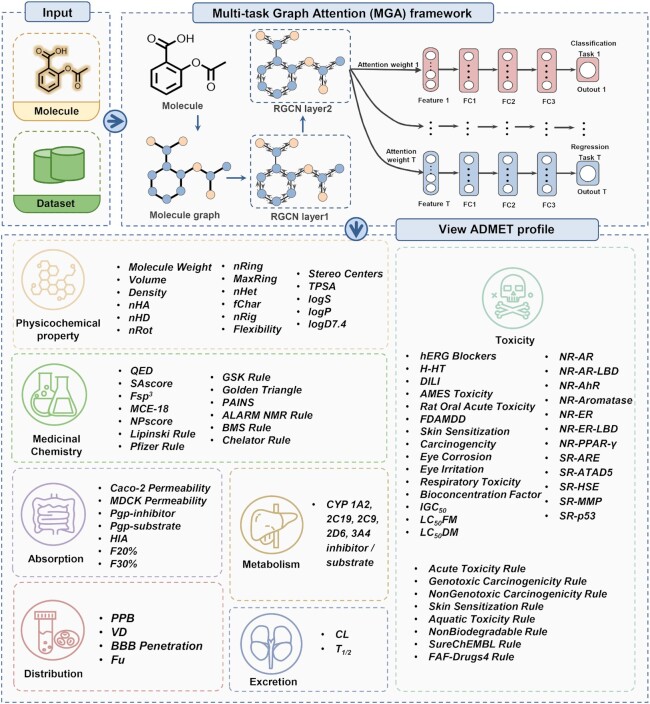
Workflow scheme of ADMETlab 2.0.

To better facilitate the ADMET evaluation of chemicals, ADMETlab 2.0 is presented with significant updates to functional modules, predictive models, and explanations. Additionally, we also updated the user interface to improve the user experience. Figure 1 presents the workflow of ADMETlab 2.0.

### Comprehensively enhanced ADMET profiles

Originally, ADMETlab could support the predictions of 7 basic physicochemical properties, 28 ADMET-related properties and 6 drug-likeness rules, but some important endpoints were still not taken into consideration. Moreover, the drug-likeness rules supported by ADMElab had overlaps between their internal terms. These problems may lead to an inadequate understanding of the whole ADMET spectrum for a molecule. Therefore, ADMETlab needs to be updated to overcome the existing limitations.

The ADME-related endpoints could be roughly divided into seven different sections: (i) physicochemical properties, (ii) medicinal chemistry properties, (iii) absorption, (iv) distribution, (v) metabolism, (vi) excretion and (vii) toxicity. Most of the newly added endpoints in ADMETlab 2.0 belong to physicochemical properties, medicinal chemistry properties and toxicity. At first, we added a number of structural properties closely relevant to drug-likeness, such as formal charge, flexibility, and stereo centers (26–28). Then, the empirical formula of logP was replaced by a well-trained MGA model based on more than ten thousand molecules. For medicinal chemistry properties, we selected 4 complementary drug-likeness rules, including Lipinski's rule-of-five (29), Pfizer rule (30), GSK rule (31) and Golden Triangle (32). These rules were born within the long-term drug discovery practice of world-famous pharmaceutical companies. Some quantitative measures, including QED (33), Fsp^3^ (34), MCE-18 (35), SAscore (36), and NPscore (37), were also added to this section to evaluate molecular drug-likeness, medicinal chemistry evolution, synthetic feasibility, and natural product likeness, respectively. Thereinto, the Fsp^3^ measure is a simple and interpretable metric for molecular saturation, and it is defined as the fraction of sp^3^ carbon atoms (the number of sp^3^ hybridized carbons / total carbon count). This parameter is closely related to solubility and melting points. The QED measure is a publicly accepted drug-likeness parameter. It is derived from a multivariate nonlinear function, in which several properties were parameterized and combined, including those used by Lipinski's rule-of-five, PSA, N_rotb_, counts of aromatic rings (N_Ar_), and the presence of certain undesirable structural motifs. We also noticed the frequent hitters (38) revoked more and more attention, and therefore four kinds of substructural rules were added to facilitate the detection of frequent hitters, thereby avoiding false positive results in drug discovery. For the toxicity section, the involvement of Tox21 made the toxicity prediction of our platform extend to biological target-based pathways. The binding data for twelve different biological targets, belonging to two major groups (the nuclear receptor pathway and the stress response pathway), constitute the complete Tox21 dataset. The prediction results of these endpoints in Tox21 could give users information on how chemicals may affect human health. In addition to traditional QSPR models, eight different toxicophore rules were integrated into this section, including human toxicity, environmental toxicity and comprehensive toxicity. It was believed that the combined application of toxicity prediction models and toxicophore rules could greatly improve the predictivity and interpretability of models (18). Other sections were also expanded in different degrees, and the introduction of the supported endpoints was provided in Supplementary Data and the website.

Further, it should be noted that the enhancement of ADMElab was not only confined to the addition of ADMET-related endpoints. The quality and quantity of the experimental data for model building was significantly improved. For example, the number of the molecules in the logD 7.4 dataset increased roughly tenfold from 1031 to 10 370, and a dramatic data increase also occurred to the hERG dataset. Other datasets were also extended with different magnitudes, such as Clearance, *T*_1/2_, VD, Caco-2 permeability, etc. Meanwhile, the number of some datasets decreased due to more rigorous data curation, where loosely defined molecules were removed and irrelevant data consolidation was corrected. Collectively, our platform could support the calculation of 88 ADMET-related endpoints spanning seven different categories, which is currently the most integrated online platform of this kind. In terms of the data, compared with the initial version, the number of the entries for model training in the current release has almost tripled.

### Re-engineered modules and batch evaluation support

In the previous release of ADMETlab, we designed three main modules: *Drug-likenessAnalysis*, *ADMET Prediction*, and *Systematic Evaluation*, whose meanings were self-explanatory. Drug-likeness analysis and ADMET evaluation were separated into different modules, and single-property prediction and all-sided ADMET prediction were both supported to meet the demands of users. Our original intention was to make the webserver well organized. However, according to the feedback from users, most people preferred to obtain the whole ADMET profile as the foundation of the decision-making that whether the chemicals were worth further exploration, rather than specific endpoints. The single-property evaluation module seemed redundant and embarrassed in most situations. Moreover, due to the absence of drug-likeness rules in Systematic Evaluation, the users had to switch back and forth between the two modules, leading to bad user experience. To solve these problems, we re-engineered and optimized the functional modules from three aspects. Firstly, the single-property prediction module was completely removed. Secondly, the independent drug-likeness module was incorporated into the Systematic Evaluation as the Medicinal Chemistry section. The combined module could provide an integral calculation and evaluation of ADMET profiles by entering molecules only once, and it was renamed to ADMET Evaluation to reflect its essence more intuitively. Further, ADMETlab also had a built-in database, which included >280 000 entries from multiple data sources, recording the basic information and ADMET profiles. In addition to accurate searching, it could implement similarity searching with the user-defined criterion and provide the most similar molecules to the query molecules. However, some public integrated databases have matured considerably in offering high-quality and up-to-date resources, such as ChEMBL, BindingDB, admetSAR, etc. Moreover, it is hard to measure to which extent similarity searching benefit the users in their structure optimization work. Considering all the factors together, the database will be no longer retained within the latest version. Nevertheless, the interested readers still could access this function in the previous version.

Another notable weakness of the previous version is the inability of batch evaluation. The application of *in silico* frameworks enables earlier ADMET evaluation, even before syntheses. It also means significantly higher computation demands, from dozens to thousands in number. Clearly, single-molecule evaluation was powerless to handle a large number of molecules unless drawing support from in-house scripts. During the past three years, there were numerous letters from different institutes asking for the possibility of adding additional batch evaluation function. As a response, we developed ADMET Screening as an independent module supporting batch uploading and downloading, which could effectively avoid laboring operations for fetching results one by one. By using this module, synthetic chemists and computational chemists could conveniently evaluate a series of empirically designed or virtually screened compounds before syntheses and biochemical assays.

In summary, ADMETlab 2.0 currently provides the users with concise, explicit, and efficient functional modules to facilitate the ADMET evaluation of chemicals.

### Robust and accurate MGA models

As the core component of the web server, the accuracy of models determines the reliability of predictions. With fixed datasets, the performance of ADMET evaluation models depends heavily on the selection of algorithms and descriptors. Traditionally, researchers needed to select multiple types of descriptors as the proxy for molecular structures, each combined with several algorithms, to construct prediction models and evaluate prediction performance, thereby obtaining the optimum combination. Such orthogonal strategy was also employed in the development of ADMETlab, where a total of 11 molecular descriptors and 6 modeling algorithms were engaged in the comparison. Although the process was tedious, a series of high-quality models with satisfactory performance were successfully constructed and embedded into the webserver.

Nevertheless, we asked ourselves: can we do better? Currently, the use of deep neural networks (DNN) has become popular throughout computational chemistry. Supported by many publications (39,40), it could obtain great improvement over classical approaches using random forest and fingerprints for many endpoints, such as solubility, Caco-2 permeability, log *D*, etc. In this work, we employed the MGA framework to develop the classification and regression predictors simultaneously for further performance improvement. MGA operated on graph-structured data, where the input molecule was regarded as a graph, with atoms being the nodes and bonds the edges. The node features were learned by propagating the features from the neighboring nodes and learning affine transformations. By learning jointly over multiple endpoints, the parameters in the hidden layers were shared among all tasks to force the learning of useful representation of the input molecule, which could improve the model generalization ability and enable tasks with fewer measures to benefit from the chemical space coverage of the larger tasks. Finally, 40 classification endpoints and 13 regression endpoints were built and evaluated in a separate test set fashion. For the classification models, the AUC ranged from 0.707 to 0.983 with an average value of 0.863. For the regression models, the *R*^2^ mainly ranged from 0.678 to 0.957 with an average value of 0.783, except for LC_50_DM. We also employed the XGBoost algorithm and MOE2d descriptors to construct traditional single-task models using the same datasets. As expected, the MGA models could yield better performance relative to the corresponding XGBoost models, except for seven classification and two regression endpoints. However, the superiority of XGBoost is quite slender (about 0.012 on AUC and 0.018 on *R*^2^) and these models may also slow down the computation speed. We still provided the users with MGA models across 53 endpoints in the web server.

Also noteworthy, the benefit brought by new algorithms is also reflected in the faster computing speed. In the MGA framework, molecules are learned in the form of graphs universally. Therefore, the laborious descriptor calculation is no longer required. Additionally, compared with the independent models for individual endpoint, multitask learning enables one input with multiple outputs, thus greatly simplifying the calculation process. Currently, a typical ADMETlab 2.0 task for 1000 molecules requires about 84 seconds, while the time required to run 20 molecules in the initial version is about 1574 s.

### Practical explanation and guidance

Unambiguous interpretation of predictions in ADMETlab had been extensively acclaimed by the scientific community. In this update, we maintained this battle-tested characteristic and made further improvement. We inherited the explicit classification of endpoints, which enabled the users to quickly find the section of interest. For example, researchers of central nervous system drugs would be particularly interested in the distribution parameters, especially blood-brain barrier penetration, which determined if the medications could have significant brain exposure for therapeutic effect. To avoid confusion, we designed different display manners. For the regression endpoints, the concrete predictive values are shown in the result page. For the classification models, the prediction probability values are represented with different symbols: 0-0.1(−−−), 0.1-0.3(−−), 0.3-0.5(−), 0.5-0.7(+), 0.7-0.9(++), and 0.9-1.0(+++). Usually, the token ‘+++’ or ‘++’ represents the molecule is more likely to be toxic or defective, while ‘−−’ or ‘−’ represents nontoxic or appropriate. Here, we do not recommend trusting predictive results symbolled by ‘+’ or ‘−' (probably values in 0.3-0.7), and corresponding molecules require further assessment. Along with the predictive results, the empirical decision state of each endpoint is visually represented with different colored dots, green for excellent, yellow for medium, and red for poor, whose definition criteria are summarized in Supplementary Data and the website. For substructural alerts, like PAINS and SureChEMBL, in addition to the alert counts, the users could check the substructures contained in the molecule through the DETAIL button. The information of each endpoint is folded in the information icon to facilitate the understanding of the predictive results, including endpoints explanation, empirical optimal range, and label definition of binary endpoints. Generally, ADMETlab 2.0 provides practical explanation and information to help the users to get a whole ADMET picture of the input molecule.

## APPLICATION CASE

To further demonstrate the reliability of ADMETlab 2.0, we selected olaparib as the input molecule to predict its ADMET parameters and to discuss the results in detail. olaparib is a poly (ADP-ribose) polymerase inhibitor developed by AstraZeneca for the treatment of BRCA mutation-positive ovarian cancer (41). As a globally approved antitumor drug, some ADMET-related parameters have been reported in several studies. The results page of Olaparib is provided in Supplementary Figure S2.

It can be seen from the radar plot that all the physicochemical properties are in the proper scope except solubility (logS). Poor solubility also affects the Fsp3 score, barely 0.33 with a red empirical decision state. In reality, its poor solubility has troubled the pharmacist for a long time and many advanced drug research techniques have been proposed to ensure its bioavailability (42). In the absorption section, olaparib is predicted as P-glycoprotein (Pgp) substrate and inhibitor with high possibility, which is consistent with the publication demonstrating that increased Pgp drug efflux transporter expression enables an intrinsic resistance of olaparib in metaplastic breast carcinoma (43). For metabolism, it is predicted that olaparib does not inhibit CYP 1A2 and CYP 2D6, and causes inhibition of CYP3A4 and CYP 2C9, which is consistent with the results of the *in vitro* cytochrome P450 evaluation reported by AstraZeneca (44). For toxicology, olaparib may have a high risk of human hepatotoxicity and liver injury, which is a common side effect of antitumor drugs and has been reported in a case study (45). Moreover, olaparib is predicted active for the ARE toxicological pathway with high possibility, although this characteristic has not been reported in any publication. Actually, antioxidant capacity has been recognized as a potential mechanism of tumor malignancy (46), and it is possible that the antitumor action of olaparib is partly contributed from the interference with the Keap1-Nrf2-ARE signaling pathway. In summary, the predictive results of ADMETlab 2.0 basically correspond to the reported experimental data, highlighting the reliability of this server tool.

### Comparison with other web-based tools

Currently, there are many online prediction servers for the evaluation of certain ADMET parameters, such as SuperCYPsPred (47) for cytochrome activity prediction, eMolTox (48) for potential toxicity prediction, ChemAGG (49) for colloidal aggregators identification, etc. Meanwhile, several excellent online platforms have been proposed for more systematic and convenient ADMET predictions, including SwissADME (14), admetSAR 2.0 (13), FAF-Drugs4 (12), pkCSM (16) and vNN-ADMET (50). Here, we compared these tools and ADMETlab 2.0, as well as its previous version, with details summarized in Table 3.

**Table 3.** Comparison of the main features of ADMETlab (version 1.0–2.0) with other web-based tools. Symbol ‘+’ means feature support and more ‘+’ indicate batter support

	ADMETlab						
Features	2.0	1.0	SwissADME	admetSAR 2.0	FAF-Drugs4	pkCSM	vNN-ADME
**Endpoints**							
Physicochemical property	17	7	12	5	20	6	0
Medicinal chemistry	13	6	10	0	16	0	0
ADME	23	21	9	35	0	20	9
Toxicity	27	7	0	12	0	10	6
Toxicophore rule	8	0	0	0	4	0	0
PAINS included	Yes	No	Yes	No	Yes	No	No
**Batch evaluation**	+++	+	+	+	++	++	++
**Explanation**	+++	++	++	+	++	++	+
**Availability**	Free	Free	Free	Free	Free	Free	Registration required
**Computation time** **(1000 molecules)**	84 s	More than 2 h	1560 s	267 s	967s	1845 s	2400 s

*Medicinal chemistry contains drug-likeness rules, chemical friendly measures, and substructural rules of frequent hitters; ADME contains absorption, distribution, metabolism, and excretion related endpoints; Toxicity contains human toxicity, animal toxicity, environmental toxicity, and toxic pathways.

**URL links:**

ADMETlab: http://admet.scbdd.com/

SwissADME: http://www.swissadme.ch/

admetSAR 2.0: http://lmmd.ecust.edu.cn/admetsar2/

FAF-Drugs4: https://fafdrugs4.rpbs.univ-paris-diderot.fr/

pkCSM: http://biosig.unimelb.edu.au/pkcsm/prediction

vNN-ADMET: https://vnnadmet.bhsai.org/vnnadmet/login.xhtml

Specifically, admetSAR, pkCSM and vNN-ADMET were designed to assist researchers in understanding the ADMET characteristics of chemicals. Thereinto, admetSAR 2.0 included the most diverse metabolism properties, while the available predictions in vNN-ADMETb were relatively limited. FAF-Drugs4 and Swiss-ADME performed outstandingly in physicochemical and medicinal chemistry properties. FAF-Drugs4 made the first attempt to add toxicophore rules into ADMET evaluation, and Swiss-ADME could provide multiple calculation approaches for logP and logS and various drug-likeness rules. However, their pharmacokinetics predictions were inferior to other tools. In comparison, ADMETlab 2.0 is available for the prediction of every important aspect of molecular quality, covering major endpoints that medicinal chemists would be interested in. Some toxicophore rules, toxicity pathways, and some medicinal chemistry measures of our platform are unique among the tools of this kind. Backed up by the robust QSPR models trained by the MGA framework, it now can provide more reliable predictions relative to other tools based on traditional machine learning algorithms. Moreover, the updated version could provide more user-friendly design and efficient service, reflected in diverse input approaches, practical explanation and high computation speed. Collectively, ADMETlab 2.0 provides medicinal chemists a comprehensive, accurate, efficient, and user-friendly service for ADMET evaluation.

## CONCLUSIONS

Here, we have introduced ADMETlab 2.0 which significantly enhanced the functionality of its predecessor. The new webserver provides the users easy access to comprehensive, accurate and efficient prediction of the ADMET profiles for chemicals, including absorption, distribution, metabolism, excretion and toxicity properties, as well as some important physicochemical and medicinal chemistry properties. In this update, the available ADMET profile is extended to 88 related characteristics, roughly twice the number of its predecessor. Meanwhile, the advanced MGA framework was employed to construct robust and accurate models as the foundation of our platform. Module restructure and batch computation support were implemented to improve usability and user experience. Optimized results explanation enables non-expert users to understand the hints behind the predicted values, thereby guiding the medicinal chemistry decision-making. We believe that ADMETlab 2.0 will prove useful in accelerating the process of drug R&D.

## Supplementary Material

gkab255_Supplemental_File
