# Supplementary material for: ADMETlab 2.0: an integrated online platform for accurate and comprehensive predictions of ADMET properties
Source: Nucleic Acids Res. 2021 Apr 24;49(W1):W5–W14. doi: 10.1093/nar/gkab255 (PMC8262709; doi:10.1093/nar/gkab255)
Supplement: gkab255_Supplemental_File [file gkab255_supplemental_file.pdf]

SUPPLEMENTARY DATA

ADMETlab 2.0: an integrated online platform for accurate and comprehensive predictions of ADMET properties

Guo-Li Xiong[1], Zhen-Xing Wu[2], Jia-Cai Yi[3], Li Fu[1], Zhi-Jiang Yang[1], Chang-Yu Hsieh[4], Ming-Zhu Yin[5], Xiang-Xiang Zeng[6], Cheng-Kun Wu[3], Ai-Ping Lu[7], Xiang Chen[5], Ting-Jun Hou[2]*, Dong-Sheng Cao[1,7]*

[1]Xiangya School of Pharmaceutical Sciences, Central South University, Changsha 410003, Hunan, P. R. China

[2]Hangzhou Institute of Innovative Medicine, College of Pharmaceutical Sciences, Zhejiang University, Hangzhou 310058, Zhejiang, P. R. China

[3]College of Computer, National University of Defense Technology, Changsha 410073, Hunan, P.R. China

[4]Tencent Quantum Laboratory, Tencent, Shenzhen 518057, Guangdong, P. R. China

[5]Department of Dermatology, Hunan Engineering Research Center of Skin Health and Disease, Hunan Key Laboratory of Skin Cancer and Psoriasis, Xiangya Hospital, Central South University, Changsha 410008, Hunan, China,

[6]Deparment of Computer Science, Hunan University, Changsha 410082, Hunan, P.R. China

[7]Institute for Advancing Translational Medicine in Bone and Joint Diseases, School of Chinese Medicine, Hong Kong Baptist University, Hong Kong SAR, China

The first three authors contribute to this paper equally.

Corresponding authors:

Ting-Jun Hou

E-mail: tingjunhou@zju.edu.cn

Tel: +86-571-88208412

Dong-Sheng Cao

Email: oriental-cds@163.com

Tel: +86-731-89824761

Table of Contents

Multi-task Graph Attention (MGA) framework 3
Explanation of available endpoints 4
1) Physicochemical property 4
2) Medicinal Chemistry 6
3) Absorption 8
4) Distribution 10
5) Metabolism 11
6) Excretion 12
7) Toxicology 12
Figure S1. An overview of the Multi-task Graph Attention framework. 21
Figure S2. The ADMET profile of olaparib. 22
Table S1. Information of the training sets, test sets, and validation sets. 23
Table S2. Scaffolds analysis of 53 datasets. 25
Table S3. Performance of classification models in training and validation sets. 26
Table S4. Performance of regression models in training and validation sets. 27
Table S5. Results of leave-cluster-out validation of classification models. 28
Table S6. Results of leave-cluster-out validation of regression models. 29

Multi-task Graph Attention (MGA) framework

An overview of the Multi-task Graph Attention framework is shown in Figure S1. As shown in **Figure S1**, MGA is composed of input, Relation graph convolution network (RGCN) layers, attention layer and fully-connected (FC) layers. In the Input, a node represents the information of an atom, and after passing RGCN layers, the node represents general features of circular substructure centered on the atom. RGCN is an extension of the standard graph convolution network (GCN) by introducing edge features to enrich the messages used to update the hidden states in the network. The propagation rule for each node v in RGCN layer is calculated via

$$h_v^{(l+1)} = \sigma\left(\sum_{r\in R} \sum_{u\epsilon N_v^r} W_r^{(l)} h_u^{(l)} + W_u^{(l)} h_v^{(l)}\right) \quad (1)$$

where $h_v^{(l+1)}$ is the state vector of target node v after $l+1$ iterations and N_v^r denotes the neighbors of node v under the relation (edge) $r\epsilon R$. $W_r^{(l)}$ is the weight for neighbor node u connecting to node v by an edge attributed with the relation $r\epsilon R,$ and $W_0^{(l)}$is the weight for target node v. As can be seen above, the edge information is explicitly incorporated in a RGCN under the relation $r\epsilon R$. The weight $W_r^{(l)}$ is a linear combination of basis transformation.

As shown in **Figure S1B**, attention layers can assign different attention weights to different substructures, and then generate the customized fingerprints (CFP) from the general features for a specific task. The attention weights and customized fingerprints are generated as follows:

$$\omega_v = \sigma(W \cdot h_v + bias) \quad (2)$$

$$CFP = \sum_{v=1}^{N}(\omega_v \cdot h_v) \quad (3)$$

where W and $bias$ are the parameters of attention layers learned in model training, *N* is the number of nodes (substructures), ω_v is the attention weight of node (substructure) v, and h_v is the general feature of node (substructure) v.

As shown in **Figure S1A**, fully-connected (FC) layers predict the corresponding tasks based on the customized toxicity fingerprints. The classification and regression tasks adopt different loss functions ($loss\_c$ and $loss\_r$) as follows:

$$loss\_c = \sum_{n=1}^{N} \sum_{c=1}^{C}(-[p_c y_{n,c} \cdot log\sigma(x_{n,c}) + (1-y_{n,c}) \cdot \log(1-\sigma(x_{n,c}))]) \quad (4)$$

$$loss\_r = \sum_{n=1}^{N} \sum_{r=1}^{R}(x_{n,r} - y_{n,r})^2 \quad (5)$$

where $x_{n,c}$ is the predict value of molecule n for classification task c, $y_{n,c}$ is the true values of molecule n for classification task c, p_c is the weight of positive samples, $x_{n,r}$ is the predict value of molecule n for regression task r, $y_{n,r}$ is the true value of molecule n for regression task r, N is the number of molecules, C is the number of the classification tasks, and R is the number of the regression tasks.

The loss function of MGA is a combination of $loss\_c$ and $loss\_r$:

$$loss = loss_c + loss\_r \quad (6)$$

Explanation of available endpoints

1) Physicochemical property

Molecular Weight

Contain hydrogen atoms. Optimal:100~600, based on Drug-Like Soft rule.

Volume

Van der Waals volume.

Density

Density = MW / Volume

nHA

Number of hydrogen bond acceptors. Sum of all O and N. Optimal: 0~12, based on Drug-Like Soft rule.

nHD

Number of hydrogen bond donors. Sum of all OHs and NHs. Optimal:0~7, based on Drug-Like Soft rule.

nRot

Number of rotatable bonds. In some situation Amide C-N bonds are not considered because of their high rotational energy barrier. Optimal:0~11, based on Drug-Like Soft rule.

nRing

Number of rings. Smallest set of smallest rings. Optimal:0~6, based on Drug-Like Soft rule.

MaxRing

Number of atoms in the biggest ring. Number of atoms involved in the biggest system ring. Optimal:0~18, based on Drug-Like Soft rule.

nHet

Number of heteroatoms. Number of non-carbon atoms (hydrogens included). Optimal:1~15, based on Drug-Like Soft rule.

fChar

Formal charge. Optimal:-4 ~4, based on Drug-Like Soft rule

nRig

Number of rigid bonds. Number of non-flexible bonds, in opposite to rotatable bonds. Optimal:0~30, based on Drug-Like Soft rule.

Flexibility

Flexibility = nRot / nRig

Stereo Centers

Number of stereocenters. Optimal: ≤ 2, based on Lead-Like Soft rule.

TPSA

Topological polar surface area. Sum of tabulated surface contributions of polar fragments. Optimal:0~140, based on *Veber* rule.

logS

- The logarithm of aqueous solubility value. The first step in the drug absorption process is the disintegration of the tablet or capsule, followed by the dissolution of the active drug. Low solubility is detrimental to good and complete oral absorption, and early measurement of this property is of great importance in drug discovery.
- Results interpretation: The predicted solubility of a compound is given as the logarithm of the molar concentration (log mol/L). Compounds in the range from -4 to 0.5 log mol/L will be considered proper.

logP

- The logarithm of the n-octanol/water distribution coefficient. log P possess a leading position with considerable impact on both membrane permeability and hydrophobic binding to macromolecules, including the target receptor as well as other proteins like plasma proteins, transporters, or metabolizing enzymes.
- Results interpretation: The predicted logP of a compound is given as the logarithm of the molar concentration (log mol/L). Compounds in the range from 0 to 3 log mol/L will be considered proper.

logD7.4

- The logarithm of the n-octanol/water distribution coefficients at pH=7.4. To exert a therapeutic effect, one drug must enter the blood circulation and then reach the site of action. Thus, an eligible drug usually needs to keep a balance between lipophilicity and hydrophilicity to dissolve in the body fluid and penetrate the biomembrane effectively. Therefore, it is important to estimate the n-octanol/water distribution coefficients at physiological pH (logD7.4) values for candidate compounds in the early stage of drug discovery.
- Results interpretation: The predicted logD7.4 of a compound is given as the logarithm of the molar concentration (log mol/L). Compounds in the range from 1 to 3 log mol/L will be considered proper.

2) Medicinal Chemistry

QED [1]

- A measure of drug-likeness based on the concept of desirability. QED is calculated by integrating the outputs of the desirability functions based on eight drug-likeness related properties, including MW, log P, N_{HBA}, N_{HBD}, PSA, N_{rotb}, the number of aromatic rings (N_{Ar}), and the number of alerts for undesirable functional groups. Here, average descriptor weights were used in the calculation of QED. The QED score is calculated by taking the geometric mean of the individual desirability functions, given by $QED = exp\left(\frac{1}{n}\sum_{i=1}^{n}\ln d_i\right)$, where d_i indicates the d_{th} desirability function and n = 8 is the number of drug-likeness related properties.
- Results interpretation: The mean QED is 0.67 for the attractive compounds, 0.49 for the unattractive compounds and 0.34 for the unattractive compounds considered too complex.
- Empirical decision: > 0.67: excellent (green); ≤ 0.67: poor (red)

SAscore [2]

- Synthetic accessibility score is designed to estimate ease of synthesis of drug-like molecules, based on a combination of fragment contributions and a complexity penalty. The score is between 1 (easy to make) and 10 (very difficult to make). The synthetic accessibility score (SAscore) is calculated as a combination of two components: $SAscore = fragmentScore - complexityPenalty$.
- Results interpretation: high SAscore: ≥ 6, difficult to synthesize; low SAscore: < 6, easy to synthesize
- Empirical decision: ≤ 6： excellent (green); > 6: poor (red)

Fsp³ [3]

- Fsp^3, the number of sp3 hybridized carbons/total carbon count, is used to determine the carbon saturation of molecules and characterize the complexity of the spatial structure of molecules. It has been demonstrated that the increased saturation measured by Fsp^3 and the number of chiral centers in the molecule increase the clinical success rate, which might be related to the increased solubility, or the fact that the enhanced 3D features allow small molecules to occupy more target space.
- Results interpretation: Fsp3 ≥ 0.42 is considered a suitable value.
- Empirical decision: ≥ 0.42： excellent (green); < 0.42: poor (red)

MCE-18 [4]

- MCE-18 stands for medicinal chemistry evolution in 2018, and this measure can effectively score molecules by novelty in terms of their cumulative sp3 complexity. It can effectively score structures by their novelty and current lead potential in contrast to simple and in many cases false positive sp3 index, and given by the following equation: $MCE18 = \left(AR + NAR + CHIRAL + SPIRO + \frac{sp^3+Cyc-Acyc}{1+sp^3}\right) \times Q^1$, where

AR is the presence of an aromatic or heteroaromatic ring (0 or 1), NAR is the presence of an aliphatic or a heteroaliphatic ring (0 or 1), CHIRAL is the presence of a chiral center (0 or 1), SPIRO is the presence of a spiro point (0 or 1), sp3 is the portion of sp3-hybridized carbon atoms (from 0 to 1), Cyc is the portion of cyclic carbons that are sp3 hybridized (from 0 to 1), Acyc is a portion of acyclic carbon atoms that are sp3 hybridized (from 0 to 1), and Q1 is the normalized quadratic index.

- Results interpretation: < 45: uninteresting, trivial, old scaffolds, low degree of 3D complexity and novelty; 45~63: sufficient novelty, basically follow the trends of currently observed in medicinal chemistry; 63~78: high structural similarity to the compounds disclosed in patent records; >78: need to be inspected visually to assess their target profile and drug-likeness.
- Empirical decision: ≥ 45： excellent (green); < 45: poor (red)

NPscore *[5]*

- The Natural Product-likeness score is a useful measure which can help to guide the design of new molecules toward interesting regions of chemical space which have been identified as “bioactive regions” by natural evolution. The calculation consists of molecule fragmentation, table lookup, and summation of fragment contributions.
- Results interpretation: The calculated score is typically in the range from −5 to 5. The higher the score is, the higher the probability is that the molecule is a NP.

Lipinski Rule *[6]*

- Content: MW≤500; logP≤5; Hacc≤10; Hdon≤5
- Results interpretation: If two properties are out of range, a poor absorption or permeability is possible, one is acceptable.
- Empirical decision: < 2 violations： excellent (green)； ≥2 violations: poor (red)

Pfizer Rule *[7]*

- Content: logP > 3; TPSA < 75
- Results interpretation: Compounds with a high log P (>3) and low TPSA (<75) are likely to be toxic.
- Empirical decision: two conditions satisfied: poor (red); otherwise: excellent (green)

GSK Rule *[8]*

- Content: MW ≤ 400; logP ≤ 4
- Results interpretation: Compounds satisfying the GSK rule may have a more favorable ADMET profile.
- Empirical decision: 0 violations: excellent (green); otherwise: poor (red)

***Golden Triangle* [9]**

- Content: 200 ≤MW ≤50; -2 ≤ logD ≤5
- Results interpretation: Compounds satisfying the GoldenTriangle rule may have a more favourable ADMET profile.
- Empirical decision: 0 violations: excellent (green); otherwise: poor (red)

***PAINS* [10]**

- Pan Assay Interference Compounds (PAINS) is one of the most famous frequent hitters filters, which comprises 480 substructures derived from the analysis of FHs determined by six target-based HTS assay. By application of these filters, it is easier to screen false positive hits and to flag suspicious compounds in screening databases. One of the most authoritative medicine magazines *Journal of Medicinal Chemistry* even requires authors to provide the screening results with the PAINS alerts of active compounds when submitting manuscripts.
- Results interpretation: If the number of alerts is not zero, the users could check the substructures by the DETIAL button.

***ALARM NMR Rule* [11]**

- Thiol reactive compounds. There are 75 substructures in this endpoint.
- Results interpretation: If the number of alerts is not zero, the users could check the substructures by the DETIAL button.

***BMS Rule* [12]**

- Undesirable, reactive compounds.There are 176 substructures in this endpoint.
- Results interpretation: If the number of alerts is not zero, the users could check the substructures by the DETIAL button.

***Chelator Rule* [13]**

- Chelating compounds. There are 55 substructures in this endpoint.
- Results interpretation: If the number of alerts is not zero, the users could check the substructures by the DETIAL button.

3) Absorption

Caco-2 Permeability

- Before an oral drug reaches the systemic circulation, it must pass through intestinal cell membranes via passive diffusion, carrier-mediated uptake or active transport processes. The human colon adenocarcinoma cell lines (Caco-2), as an alternative approach for the human intestinal epithelium, has

been commonly used to estimate in vivo drug permeability due to their morphological and functional similarities. Thus, Caco-2 cell permeability has also been an important index for an eligible candidate drug compound.

- Results interpretation: The predicted Caco-2 permeability of a given compound is given as the log cm/s. A compound is considered to have a proper Cao-2 permeability if it has predicted value >-5.15log cm/s.
- Empirical decision: > -5.15: excellent (green); otherwise: poor (red)

MDCK Permeability

- Madin−Darby Canine Kidney cells (MDCK) have been developed as an in vitro model for permeability screening. Its apparent permeability coefficient, P_{app}, is widely considered to be the in vitro gold standard for assessing the uptake efficiency of chemicals into the body. P_{app} values of MDCK cell lines are also used to estimate the effect of the blood-brain barrier (BBB).
- Results interpretation: The unit of predicted MDCK permeability is cm/s. A compound is considered to have a high passive MDCK permeability for a $P_{app} > 20 \times 10^{-6}$ cm/s, medium permeability for 2-20 x 10^{-6}cm/s, low permeability for < 2 x 10^{-6}cm/s.
- Empirical decision: >2 x 10^{-6}cm/s: excellent (green), otherwise: poor (red)

Pgp-inhibitor

- The inhibitor of P-glycoprotein. The P-glycoprotein, also known as MDR1 or 2 ABCB1, is a membrane protein member of the ATP-binding cassette (ABC) transporters superfamily. It is probably the most promiscuous efflux transporter, since it recognizes a number of structurally different and apparently unrelated xenobiotics; notably, many of them are also CYP3A4 substrates.
- Results interpretation: Category 0: Non-inhibitor; Category 1: Inhibitor. The output value is the probability of being Pgp-inhibitor, within the range of 0 to 1.
- Empirical decision: 0-0.3: excellent (green); 0.3-0.7: medium (yellow); 0.7-1.0(++): poor (red)

Pgp-substrate

- As described in the Pgp-inhibitor section, modulation of P-glycoprotein mediated transport has significant pharmacokinetic implications for Pgp substrates, which may either be exploited for specific therapeutic advantages or result in contraindications.
- Results interpretation: Category 0: Non-substrate; Category 1: substrate. The output value is the probability of being Pgp-substrate, within the range of 0 to 1.
- Empirical decision: 0-0.3: excellent (green); 0.3-0.7: medium (yellow); 0.7-1.0(++): poor (red)

HIA

- Human intestinal absorption. As described above, the human intestinal absorption of an oral drug is the essential prerequisite for its apparent efficacy. What's more, the close relationship between oral bioavailability and intestinal absorption has also been proven and HIA can be seen an alternative indicator

for oral bioavailability to some extent.

- Result interpretation: A molecule with an absorbance of less than 30% is considered to be poorly absorbed. Accordingly, molecules with a HIA >30% were classified as HIA- (Category 0), while molecules with a HIA < 30% were classified as HIA+(Category 1). The output value is the probability of being HIA+, within the range of 0 to 1.
- Empirical decision: 0-0.3: excellent (green); 0.3-0.7: medium (yellow); 0.7-1.0(++): poor (red)

$F_{20\%}$

- The human oral bioavailability 20%. For any drug administrated by the oral route, oral bioavailability is undoubtedly one of the most important pharmacokinetic parameters because it is the indicator of the efficiency of the drug delivery to the systemic circulation.
- Result interpretation: Molecules with a bioavailability ≥ 20% were classified as $F_{20\%}$- (Category 0), while molecules with a bioavailability < 20% were classified as $F_{20\%}$+ (Category 1). The output value is the probability of being $F_{20\%}$+, within the range of 0 to 1.
- Empirical decision: 0-0.3: excellent (green); 0.3-0.7: medium (yellow); 0.7-1.0(++): poor (red)

$F_{30\%}$

- The human oral bioavailability 30%. For any drug administrated by the oral route, oral bioavailability is undoubtedly one of the most important pharmacokinetic parameters because it is the indicator of the efficiency of the drug delivery to the systemic circulation.
- Result interpretation: Molecules with a bioavailability ≥ 30% were classified as $F_{30\%}$- (Category 0), while molecules with a bioavailability < 30% were classified as $F_{30\%}$+ (Category 1). The output value is the probability of being $F_{30\%}$+, within the range of 0 to 1.
- Empirical decision: 0-0.3: excellent (green); 0.3-0.7: medium (yellow); 0.7-1.0(++): poor (red)

4) Distribution

PPB

- Plasma protein binding. One of the major mechanisms of drug uptake and distribution is through PPB, thus the binding of a drug to proteins in plasma has a strong influence on its pharmacodynamic behavior. PPB can directly influence the oral bioavailability because the free concentration of the drug is at stake when a drug binds to serum proteins in this process.
- Result interpretation: A compound is considered to have a proper PPB if it has predicted value < 90%, and drugs with high protein-bound may have a low therapeutic index.
- Empirical decision: ≤ 90%: excellent (green); otherwise: poor (red).

VD

- Volume Distribution. The VD is a theoretical concept that connects the administered dose with the actual initial concentration present in the circulation and it is an important parameter to describe the in vivo distribution for drugs. In practical, we can speculate the distribution characters for an unknown compound according to its VD value, such as its condition binding to plasma protein, its distribution amount in body fluid and its uptake amount in tissues.
- Result interpretation: The unit of predicted VD is L/kg. A compound is considered to have a proper VD if it has predicted VD in the range of 0.04-20L/kg.
- Empirical decision: 0.04-20: excellent (green); otherwise: poor (red)

BBB Penetration

- Drugs that act in the CNS need to cross the blood–brain barrier (BBB) to reach their molecular target. By contrast, for drugs with a peripheral target, little or no BBB penetration might be required in order to avoid CNS side effects.
- Result interpretation: The unit of BBB penetration is cm/s. Molecules with logBB > -1 were classified as BBB+ (Category 1), while molecules with logBB ≤ -1 were classified as BBB- (Category 0). The output value is the probability of being BBB+, within the range of 0 to 1.
- Empirical decision: 0-0.3: excellent (green); 0.3-0.7: medium (yellow); 0.7-1.0(++): poor (red)

Fu

- The fraction unbound in plasms. Most drugs in plasma will exist in equilibrium between either an unbound state or bound to serum proteins. Efficacy of a given drug may be affect by the degree to which it binds proteins within blood, as the more that is bound the less efficiently it can traverse cellular membranes or diffuse.
- Result interpretation: >20%: High Fu; 5-20%: medium Fu; <5% low Fu.
- Empirical decision: ≥ 5%: excellent (green);< 5%: poor (red).

5) Metabolism

CYP 1A2 / 2C19 / 2C9 / 2D6 / 3A4 inhibitor

CYP 1A2 / 2C19 /2C9 / 2D6 / 3A4 substrate

- Based on the chemical nature of biotransformation, the process of drug metabolism reactions can be divided into two broad categories: phase I (oxidative reactions) and phase II (conjugative reactions). The human cytochrome P450 family (phase I enzymes) contains 57 isozymes and these isozymes metabolize approximately two-thirds of known drugs in human with 80% of this attribute to five isozymes—1A2, 3A4, 2C9, 2C19 and 2D6. Most of these CYPs responsible for phase I reactions are concentrated in the liver.
- Result interpretation: Category 0: Non-substrate / Non-inhibitor; Category 1: substrate / inhibitor. The output value is the probability of being substrate / inhibitor, within the range of 0 to 1.

6) Excretion

CL

- The clearance of a drug. Clearance is an important pharmacokinetic parameter that defines, together with the volume of distribution, the half-life, and thus the frequency of dosing of a drug.
- Result interpretation: The unit of predicted CL penetration is ml/min/kg. >15 ml/min/kg: high clearance; 5-15 ml/min/kg: moderate clearance; <5 ml/min/kg: low clearance.
- Empirical decision: ≥ 5: excellent (green);< 5: poor (red).

$T_{1/2}$

- The half-life of a drug is a hybrid concept that involves clearance and volume of distribution, and it is arguably more appropriate to have reliable estimates of these two properties instead.
- Result interpretation: Molecules with $T_{1/2}$ > 3 were classified as $T_{1/2}$ - (Category 0), while molecules with $T_{1/2}$ ≤ 3 were classified as $T_{1/2}$ + (Category 1). The output value is the probability of being $T_{1/2}$+, within the range of 0 to 1.
- Empirical decision: 0-0.3: excellent (green); 0.3-0.7: medium (yellow); 0.7-1.0(++): poor (red)

7) Toxicology

hERG Blockers

- The human ether-a-go-go related gene. The During cardiac depolarization and repolarization, a voltage-gated potassium channel encoded by hERG plays a major role in the regulation of the exchange of cardiac action potential and resting potential. The hERG blockade may cause long QT syndrome (LQTS), arrhythmia, and Torsade de Pointes (TdP), which lead to palpitations, fainting, or even sudden death.
- Result interpretation: Molecules with IC_{50} more than 10 μM or less than 50% inhibition at 10 μM were classified as hERG - (Category 0), while molecules with IC_{50} less than 10 μM or more than 50% inhibition at 10 μM were classified as hERG+ (Category 1). The output value is the probability of being hERG+, within the range of 0 to 1.
- Empirical decision: 0-0.3: excellent (green); 0.3-0.7: medium (yellow); 0.7-1.0(++): poor (red)

H-HT

- The human hepatotoxicity. Drug induced liver injury is of great concern for patient safety and a major cause for drug withdrawal from the market. Adverse hepatic effects in clinical trials often lead to a late and costly termination of drug development programs.
- Result interpretation: Category 0: H-HT negative(-); Category 1: H-HT positive(+). The output value is the probability of being toxic, within the range of 0 to 1.
- Empirical decision: 0-0.3: excellent (green); 0.3-0.7: medium (yellow); 0.7-1.0(++): poor (red)

DILI

- Drug-induced liver injury (DILI) has become the most common safety problem of drug withdrawal from the market over the past 50 years.
- Result interpretation: Category 0: DILI negative(-); Category 1: DILI positive(+). The output value is the probability of being toxic, within the range of 0 to 1.
- Empirical decision: 0-0.3: excellent (green); 0.3-0.7: medium (yellow); 0.7-1.0(++): poor (red)

AMES Toxicity

- The Ames test for mutagenicity. The mutagenic effect has a close relationship with the carcinogenicity, and it is the most widely used assay for testing the mutagenicity of compounds.
- Result interpretation: Category 0: AMES negative(-); Category 1: AMES positive(+). The output value is the probability of being toxic, within the range of 0 to 1.
- Empirical decision: 0-0.3: excellent (green); 0.3-0.7: medium (yellow); 0.7-1.0(++): poor (red)

Rat Oral Acute Toxicity

- Determination of acute toxicity in mammals (e.g. rats or mice) is one of the most important tasks for the safety evaluation of drug candidates.
- Result interpretation: Category 0: low-toxicity, > 500 mg/kg; Category 1: high-toxicity; < 500 mg/kg. The output value is the probability of being toxic, within the range of 0 to 1.
- Empirical decision: 0-0.3: excellent (green); 0.3-0.7: medium (yellow); 0.7-1.0(++): poor (red)

FDAMDD

- The maximum recommended daily dose provides an estimate of the toxic dose threshold of chemicals in humans.
- Result interpretation: Category 1: FDAMDD positive(+), ≤ 0.011 mmol/kg -bw/day; Category 0: FDAMDD negative(-), > 0.011 mmol/kg-bw/day. The output value is the probability of being toxic, within the range of 0 to 1.
- Empirical decision: 0-0.3: excellent (green); 0.3-0.7: medium (yellow); 0.7-1.0(++): poor (red)

Skin Sensitization

- Skin sensitization is a potential adverse effect for dermally applied products. The evaluation of whether a compound, that may encounter the skin, can induce allergic contact dermatitis is an important safety concern.
- Result interpretation: Category 1: Sensitizer; Category 0: Non-sensitizer. The output value is the probability of being toxic, within the range of 0 to 1.
- Empirical decision: 0-0.3: excellent (green); 0.3-0.7: medium (yellow); 0.7-1.0(++): poor (red)

Carcinogencity

- Among various toxicological endpoints of chemical substances, carcinogenicity is of great concern because of its serious effects on human health. The carcinogenic mechanism of chemicals may be due to their ability to damage the genome or disrupt cellular metabolic processes. Many approved drugs have been identified as carcinogens in humans or animals and have been withdrawn from the market.
- Result interpretation: Category 1: carcinogens; Category 0: non-carcinogens. Chemicals are labelled as active (carcinogens) or inactive (non-carcinogens) according to their TD_{50} values. The output value is the probability of being toxic, within the range of 0 to 1.
- Empirical decision: 0-0.3: excellent (green); 0.3-0.7: medium (yellow); 0.7-1.0(++): poor (red)

Eye Corrosion / Irritation

- Assessing the eye irritation/corrosion (EI/EC) potential of a chemical is a necessary component of risk assessment. Cornea and conjunctiva tissues comprise the anterior surface of the eye, and hence cornea and conjunctiva tissues are directly exposed to the air and easily suffer injury by chemicals. There are several substances, such as chemicals used in manufacturing, agriculture and warfare, ocular pharmaceuticals, cosmetic products, and household products, that can cause EI or EC.
- Result interpretation: Category 1: corrosives / irritants chemicals; Category 0: non-corrosives / non-irritants chemicals. The output value is the probability of being toxic, within the range of 0 to 1.
- Empirical decision: 0-0.3: excellent (green); 0.3-0.7: medium (yellow); 0.7-1.0(++): poor (red)

Respiratory Toxicity

- Among these safety issues, respiratory toxicity has become the main cause of drug withdrawal. Drug-induced respiratory toxicity is usually underdiagnosed because it may not have distinct early signs or symptoms in common medications and can occur with significant morbidity and mortality.Therefore, careful surveillance and treatment of respiratory toxicity is of great importance.
- Result interpretation: Category 1: respiratory toxicants; Category 0: non-respiratory toxicants. The output value is the probability of being toxic, within the range of 0 to 1.
- Empirical decision: 0-0.3: excellent (green); 0.3-0.7: medium (yellow); 0.7-1.0(++): poor (red)

Bioconcentration Factor

- The bioconcentration factor BCF is defined as the ratio of the chemical concentration in biota as a result of absorption via the respiratory surface to that in water at steady state. It is used for considering secondary poisoning potential and assessing risks to human health via the food chain. The unit of BCF is log10(L/kg).

IGC_{50}

- 48 hour Tetrahymena pyriformis IGC_{50} (concentration of the test chemical in water in mg/L that causes 50% growth inhibition to Tetrahymena pyriformis after 48 hours). The unit of IGC_{50} is −log10[(mg/L)/(1000*MW)].

LC_{50}FM

- 96 hour fathead minnow LC_{50} (concentration of the test chemical in water in mg/L that causes 50% of fathead minnow to die after 96 hours). The unit of LC_{50}FM is −log10[(mg/L)/(1000*MW)].

LC_{50}DM

- 48 hour Daphnia magna LC_{50} (concentration of the test chemical in water in mg/L that causes 50% of Daphnia magna to die after 48 hours). The unit of LC_{50}DM is −log10[(mg/L)/(1000*MW)].

NR-AR

- Androgen receptor (AR), a nuclear hormone receptor, plays a critical role in AR-dependent prostate cancer and other androgen related diseases. Endocrine disrupting chemicals (EDCs) and their interactions with steroid hormone receptors like AR may cause disruption of normal endocrine function as well as interfere with metabolic homeostasis, reproduction, developmental and behavioral functions.
- Result interpretation: Category 1: actives ; Category 0: inactives. The output value is the probability of being AR agonists, within the range of 0 to 1.
- Empirical decision: 0-0.3: excellent (green); 0.3-0.7: medium (yellow); 0.7-1.0(++): poor (red)

NR-AR-LBD

- Androgen receptor (AR), a nuclear hormone receptor, plays a critical role in AR-dependent prostate cancer and other androgen related diseases. Endocrine disrupting chemicals (EDCs) and their interactions with steroid hormone receptors like AR may cause disruption of normal endocrine function as well as interfere with metabolic homeostasis, reproduction, developmental and behavioral functions.
- Result interpretation: Category 1: actives ; Category 0: inactives. Molecules that labeled 1 in this bioassay may bind to the LBD of androgen receptor. The output value is the probability of being actives, within the range of 0 to 1.
- Empirical decision: 0-0.3: excellent (green); 0.3-0.7: medium (yellow); 0.7-1.0(++): poor (red)

NR-AhR

- The Aryl hydrocarbon Receptor (AhR), a member of the family of basic helix-loop-helix transcription factors, is crucial to adaptive responses to environmental changes. AhR mediates cellular responses to environmental pollutants such as aromatic hydrocarbons through induction of phase I and II enzymes but also interacts with other nuclear receptor signaling pathways.
- Result interpretation: Category 1: actives ; Category 0: inactives. Molecules that labeled 1 may activate the aryl hydrocarbon receptor signaling pathway. The output value is the probability of being actives, within the range of 0 to 1.
- Empirical decision: 0-0.3: excellent (green); 0.3-0.7: medium (yellow); 0.7-1.0(++): poor (red)

NR-Aromatase

- Endocrine disrupting chemicals (EDCs) interfere with the biosynthesis and normal functions of steroid hormones including estrogen and androgen in the body. Aromatase catalyzes the conversion of androgen to estrogen and plays a key role in maintaining the androgen and estrogen balance in many of the EDC-sensitive organs.
- Result interpretation: Category 1: actives ; Category 0: inactives. Molecules that labeled 1 are regarded as aromatase inhibitors that could affect the balance between androgen and estrogen. The output value is the probability of being actives, within the range of 0 to 1.
- Empirical decision: 0-0.3: excellent (green); 0.3-0.7: medium (yellow); 0.7-1.0(++): poor (red)

NR-ER

- Estrogen receptor (ER), a nuclear hormone receptor, plays an important role in development, metabolic homeostasis and reproduction. Endocrine disrupting chemicals (EDCs) and their interactions with steroid hormone receptors like ER causes disruption of normal endocrine function. Therefore, it is important to understand the effect of environmental chemicals on the ER signaling pathway.
- Result interpretation: Category 1: actives ; Category 0: inactives. The output value is the probability of being actives within the range of 0 to 1.
- Empirical decision: 0-0.3: excellent (green); 0.3-0.7: medium (yellow); 0.7-1.0(++): poor (red)

NR-ER-LBD

- Estrogen receptor (ER), a nuclear hormone receptor, plays an important role in development, metabolic homeostasis and reproduction. Two subtypes of ER, ER-alpha and ER-beta have similar expression patterns with some uniqueness in both types. Endocrine disrupting chemicals (EDCs) and their interactions with steroid hormone receptors like ER causes disruption of normal endocrine function.
- Result interpretation: Category 1: actives ; Category 0: inactives. The output value is the probability of being actives within the range of 0 to 1.
- Empirical decision: 0-0.3: excellent (green); 0.3-0.7: medium (yellow); 0.7-1.0(++): poor (red)

NR-PPAR-gamma

- The peroxisome proliferator-activated receptors (PPARs) are lipid-activated transcription factors of the nuclear receptor superfamily with three distinct subtypes namely PPAR alpha, PPAR delta (also called PPAR beta) and PPAR gamma (PPARg). All these subtypes heterodimerize with Retinoid X receptor (RXR) and these heterodimers regulate transcription of various genes. PPAR-gamma receptor (glitazone receptor) is involved in the regulation of glucose and lipid metabolism.
- Result interpretation: Category 1: actives ; Category 0: inactives. The output value is the probability of being actives within the range of 0 to 1.
- Empirical decision: 0-0.3: excellent (green); 0.3-0.7: medium (yellow); 0.7-1.0(++): poor (red)

SR-ARE

- Oxidative stress has been implicated in the pathogenesis of a variety of diseases ranging from cancer to neurodegeneration. The antioxidant response element (ARE) signaling pathway plays an important role in the amelioration of oxidative stress. The CellSensor ARE-bla HepG2 cell line (Invitrogen) can be used for analyzing the Nrf2/antioxidant response signaling pathway. Nrf2 (NF-E2-related factor 2) and Nrf1 are transcription factors that bind to AREs and activate these genes.
- Result interpretation: Category 1: actives ; Category 0: inactives. The output value is the probability of being actives within the range of 0 to 1.
- Empirical decision: 0-0.3: excellent (green); 0.3-0.7: medium (yellow); 0.7-1.0(++): poor (red)

SR-ATAD5

- ATPase family AAA domain-containing protein 5. As cancer cells divide rapidly and during every cell division they need to duplicate their genome by DNA replication. The failure to do so results in the cancer cell death. Based on this concept, many chemotherapeutic agents were developed but have limitations such as low efficacy and severe side effects etc. Enhanced Level of Genome Instability Gene 1 (ELG1; human ATAD5) protein levels increase in response to various types of DNA damage.
- Result interpretation: Category 1: actives ; Category 0: inactives. The output value is the probability of being actives within the range of 0 to 1.
- Empirical decision: 0-0.3: excellent (green); 0.3-0.7: medium (yellow); 0.7-1.0(++): poor (red)

SR-HSE

- Heat shock factor response element. Various chemicals, environmental and physiological stress conditions may lead to the activation of heat shock response/ unfolded protein response (HSR/UPR). There are three heat shock transcription factors (HSFs) (HSF-1, -2, and -4) mediating transcriptional regulation of the human HSR.
- Result interpretation: Category 1: actives ; Category 0: inactives. The output value is the probability of being actives within the range of 0 to 1.
- Empirical decision: 0-0.3: excellent (green); 0.3-0.7: medium (yellow); 0.7-1.0(++): poor (red)

SR-MMP

- Mitochondrial membrane potential (MMP), one of the parameters for mitochondrial function, is generated by mitochondrial electron transport chain that creates an electrochemical gradient by a series of redox reactions. This gradient drives the synthesis of ATP, a crucial molecule for various cellular processes. Measuring MMP in living cells is commonly used to assess the effect of chemicals on mitochondrial function; decreases in MMP can be detected using lipophilic cationic fluorescent dyes.
- Result interpretation: Category 1: actives ; Category 0: inactives. The output value is the probability of being actives within the range of 0 to 1.
- Empirical decision: 0-0.3: excellent (green); 0.3-0.7: medium (yellow); 0.7-1.0(++): poor (red)

SR-p53

- p53, a tumor suppressor protein, is activated following cellular insult, including DNA damage and other cellular stresses. The activation of p53 regulates cell fate by inducing DNA repair, cell cycle arrest, apoptosis, or cellular senescence. The activation of p53, therefore, is a good indicator of DNA damage and other cellular stresses.
- Result interpretation: Category 1: actives ; Category 0: inactives. The output value is the probability of being actives within the range of 0 to 1.
- Empirical decision: 0-0.3: excellent (green); 0.3-0.7: medium (yellow); 0.7-1.0(++): poor (red)

Acute Toxicity Rule

- Molecules containing these substructures may cause acute toxicity during oral administration. There are 20 substructures in this endpoint.
- Results interpretation: If the number of alerts is not zero, the users could check the substructures by the DETIAL button.

Genotoxic Carcinogenicity Rule

- Molecules containing these substructures may cause carcinogenicity or mutagenicity through genotoxic mechanisms.There are 117 substructures in this endpoint.
- Results interpretation: If the number of alerts is not zero, the users could check the substructures by the DETIAL button.

NonGenotoxic Carcinogenicity Rule

- Molecules containing these substructures may cause carcinogenicity through nongenotoxic mechanisms. There are 23 substructures in this endpoint.
- Results interpretation: If the number of alerts is not zero, the users could check the substructures by the DETIAL button.

Skin Sensitization Rule

- Molecules containing these substructures may cause skin irritation.There are 155 substructures in this endpoint. Molecules containing these substructures may cause skin irritation.
- Results interpretation: If the number of alerts is not zero, the users could check the substructures by the DETIAL button.

Aquatic Toxicity Rule

- Molecules containing these substructures may cause toxicity to liquid(water). There are 99 substructures in this endpoint.

- Results interpretation: If the number of alerts is not zero, the users could check the substructures by the DETIAL button.

NonBiodegradable Rule

- Molecules containing these substructures may be non-biodegradable. There are 19 substructures in this endpoint.
- Results interpretation: If the number of alerts is not zero, the users could check the substructures by the DETIAL button.

SureChEMBL Rule

- Molecules matching one or more structural alerts are considered to have MedChem unfriendly status. There are 164 substructures in this endpoint.
- Results interpretation: If the number of alerts is not zero, the users could check the substructures by the DETIAL button.

FAF-Drugs4 Rule

- Molecules containing these substructures may be toxic. There are 154 substructures collected form FAF-Drugs4 webserver in this endpoint.

  Results interpretation: If the number of alerts is not zero, the users could check the substructures by the DETIAL button.

[1] Bickerton G R, Paolini G V, Besnard J, et al. Quantifying the chemical beauty of drugs[J]. Nat Chem, 2012, 4(2): 90-8.

[2] Ertl P, Schuffenhauer A. Estimation of synthetic accessibility score of drug-like molecules based on molecular complexity and fragment contributions[J]. J Cheminform, 2009, 1(1): 8.

[3] Lovering F, Bikker J, Humblet C. Escape from flatland: increasing saturation as an approach to improving clinical success[J]. J Med Chem, 2009, 52(21): 6752-6.

[4] Ivanenkov Y A, Zagribelnyy B A, Aladinskiy V A. Are We Opening the Door to a New Era of Medicinal Chemistry or Being Collapsed to a Chemical Singularity?[J]. J Med Chem, 2019, 62(22): 10026-10043.

[5] Ertl P, Roggo S, Schuffenhauer A. Natural product-likeness score and its application for prioritization of compound libraries[J]. J Chem Inf Model, 2008, 48(1): 68-74.

[6] Lipinski C A, Lombardo F, Dominy B W, et al. Experimental and computational approaches to estimate solubility and permeability in drug discovery and development settings[J]. Adv Drug Deliv Rev, 2001, 46(1-3): 3-26.

[7] Hughes J D, Blagg J, Price D A, et al. Physiochemical drug properties associated with in vivo toxicological outcomes[J]. Bioorg Med Chem Lett, 2008, 18(17): 4872-5.

[8] Gleeson M P. Generation of a set of simple, interpretable ADMET rules of thumb[J]. J Med Chem, 2008, 51(4): 817-34.

[9] Johnson T W, Dress K R, Edwards M. Using the Golden Triangle to optimize clearance and oral absorption[J]. Bioorg Med Chem Lett, 2009, 19(19): 5560-4.

[10] Baell J B, Holloway G A. New substructure filters for removal of pan assay interference compounds (PAINS) from screening libraries and for their exclusion in bioassays[J]. J Med Chem, 2010, 53(7): 2719-40.

[11] Huth J R, Mendoza R, Olejniczak E T, et al. ALARM NMR: a rapid and robust experimental method to detect reactive false positives in biochemical screens[J]. J Am Chem Soc, 2005, 127(1): 217-24.
[12] Pearce B C, Sofia M J, Good A C, et al. An empirical process for the design of high-throughput screening deck filters[J]. J Chem Inf Model, 2006, 46(3): 1060-8.
[13] Agrawal A, Johnson S L, Jacobsen J A, et al. Chelator fragment libraries for targeting metalloproteinases[J]. ChemMedChem, 2010, 5(2): 195-9

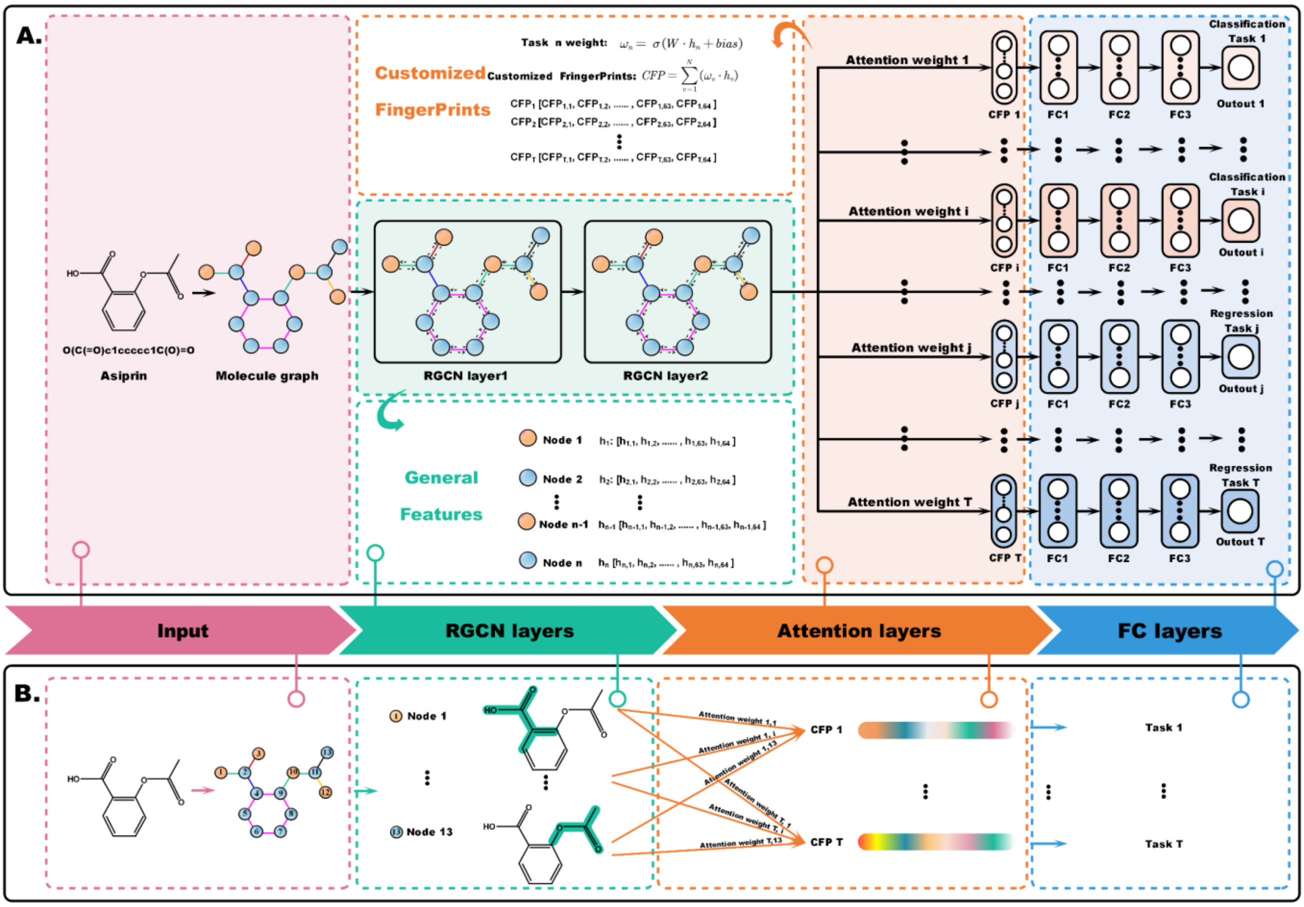

Figure S1. An overview of the Multi-task Graph Attention framework.

ADMETlab 2.0 — Home | Services | Resources | Explanation | Publications | Contact

Home / ADMET Evaluation / Evaluation Results

O=C(c1cc(Cc2n[nH]c(=O)c3ccccc23)ccc1F)N1CCN(C(=O)C2CC2)CC1 — Download as CSV | Download as PDF

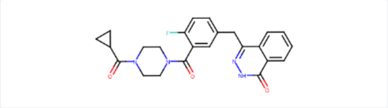

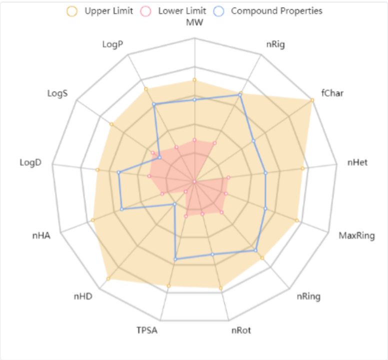

Physicochemical Property

Molecular Weight (MW)	434.180
Volume	430.939
Density	1.008
nHA	7
nHD	1
nRot	6
nRing	5
MaxRing	10
nHet	8
fChar	0
nRig	29
Flexibility	0.207
Stereo Centers	0
TPSA	86.370
logS	-4.750
logP	2.223
logD	2.183

Medicinal Chemistry

QED	0.683
SAscore	2.369
Fsp^3	0.333
MCE-18	65.812
NPscore	-1.608
Lipinski Rule	Accepted
Pfizer Rule	Accepted
GSK Rule	Rejected
Golden Triangle	Accepted
PAINS	0 alert(s)
ALARM NMR Rule	0 alert(s)
BMS Rule	0 alert(s)
Chelator Rule	0 alert(s)

Absorption

Caco-2 Permeability	-5.195
MDCK Permeability	1.2e-05
Pgp-inhibitor	+++
Pgp-substrate	+++
HIA	--
$F_{20\%}$	---
$F_{30\%}$	---

Distribution

PPB	92.949%
VD	1.310
BBB Penetration	--
Fu	4.408%

Metabolism

CYP1A2 inhibitor	---
CYP1A2 substrate	--
CYP2C19 inhibitor	+
CYP2C19 substrate	--
CYP2C9 inhibitor	++
CYP2C9 substrate	+++
CYP2D6 inhibitor	---
CYP2D6 substrate	++
CYP3A4 inhibitor	++
CYP3A4 substrate	+

Excretion

CL	3.522
$T_{1/2}$	0.306

Toxicity

hERG Blockers	-
H-HT	+++
DILI	++
AMES Toxicity	---
Rat Oral Acute Toxicity	+++
FDAMDD	-
Skin Sensitization	---
Carcinogencity	--
Eye Corrosion	---
Eye Irritation	---
Respiratory Toxicity	--
**Environmental Toxicity**	
Bioconcentration Factors	0.486
IGC_{50}	2.268
$LC_{50}FM$	2.976
$LC_{50}DM$	5.327
**Tox21 Pathway**	
NR-AR	-
NR-AR-LBD	--
NR-AhR	--
NR-Aromatase	---
NR-ER	--
NR-ER-LBD	---
NR-PPAR-gamma	-
SR-ARE	++
SR-ATAD5	---
SR-HSE	---
SR-MMP	---
SR-p53	---
**Toxicophore Rules**	
Acute Toxicity Rule	0 alert(s)
Genotoxic Carcinogenicity Rule	0 alert(s)
NonGenotoxic Carcinogenicity Rule	1 alert(s) DETAIL
Skin Sensitization Rule	0 alert(s)
Aquatic Toxicity Rule	1 alert(s) DETAIL
NonBiodegradable Rule	1 alert(s) DETAIL
SureChEMBL Rule	0 alert(s)
FAF-Drugs4 Rule	0 alert(s)

Tip: For the classification endpoints , the prediction probability values are transformed into six symbols: 0-0.1(---), 0.1-0.3(--), 0.3-0.5(-), 0.5-0.7(+), 0.7-0.9(++), and 0.9-1.0(+++).

Figure S2. The ADMET profile of olaparib.

Table S1. Information of the training sets, test sets, and validation sets

Properties	Total (positive/Negative)	training set (positive/Negative)	test set (positive/Negative)	valuation set (positive/Negative)
**LogS**	4797	3836	480	481
**LogD7.4**	10370	8296	1036	1038
**LogP**	12682	10145	1270	1267
**Caco-2 Permeability**	2464	1970	247	247
**MDCK Permeability**	1140	912	114	114
**Pgp-inhibitor**	2209 (1315/894)	1764 (1051/713)	222 (132/90)	223 (132/91)
**pgp-substrate**	1185 (586/599)	949 (471/478)	118 (58/60)	118 (57/61)
**HIA**	1160 (1022/138)	927 (818/109)	116 (101/15)	117 (103/14)
**$F_{20\%}$**	992 (753/239)	794 (602/192)	98 (75/23)	100 (76/24)
**$F_{30\%}$**	992 (666/326)	793 (532/261)	99 (67/32)	100 (67/33)
**PPB**	4712	3771	479	480
**VD**	1086	872	107	107
**BBB Penetration**	2865 (1651/1254)	2324 (1321/1003)	290 (165/125)	291 (165/126)
**Fu**	2575	2059	258	258
**CYP1A2 inhibitor**	12635 (5876/6759)	10111 (4702/5425)	1261 (588/673)	1263 (586/677)
**CYP1A2 substrate**	366 (176/190)	292 (140/152)	37 (18/19)	37 (18/19)
**CYP2C19 inhibitor**	12611 (5770/6841)	10096 (4618/5478)	1257 (577/680)	1258 (575/683)
**CYP2C19 substrate**	258 (107/151)	206 (85/121)	26 (11/15)	26 (11/15)
**CYP2C9 inhibitor**	12111 (4017/8094)	9686 (3213/6473)	1213 (402/811)	1212 (402/810)
**CYP2C9 substrate**	811 (325/486)	647 (259/388)	82 (33/49)	82 (33/49)
**CYP2D6 inhibitor**	13073 (2535/10538)	10471 (2032/8439)	1304 (255/1051)	1298 (250/1048)
**CYP2D6 substrate**	877 (435/442)	703 (347/356)	85 (44/41)	89 (44/45)
**CYP3A4 inhibitor**	12339 (5092/7247)	9880 (4074/5806)	1232 (510/722)	1227 (508/719)
**CYP3A4 substrate**	979 (497/482)	786 (397/389)	97 (49/48)	96 (51/45)
**CL**	831	666	81	84
**$T_{1/2}$**	1219 (500/719)	973 (399/574)	124 (51/73)	122 (50/72)
**hERG Blockers**	13845 (6922/6923)	11076 (5538/5538)	1384 (692/692)	1385 (692/693)
**H-HT**	2304 (1299/1005)	1850 (1044/806)	227 (128/99)	227 (127/100)
**DILI**	467 (235/232)	373 (187/186)	47 (24/23)	47 (24/23)
**AMES Toxicity**	7575 (4222/3353)	6071 (3389/2682)	751 (416/335)	753 (417/336)
**Rat Oral Acute Toxicity**	7327 (2799/4528)	5862 (2240/3622)	733 (280/453)	732 (279/453)
**FDAMDD**	1197 (561/636)	957 (448/509)	120 (56/64)	120 (57/63)
**Skin Sensitization**	405 (274/131)	324 (219/105)	40 (27/13)	41 (28/13)
**Carcinogencity**	1041 (516/525)	832 (413/419)	104 (51/53)	105 (52/53)

**Bioconcentration Factor**	676	540	68	68
**IGC_{50}**	1787	1429	179	179
**LC_{50}FM**	816	652	82	82
**LC_{50}DM**	347	277	35	35
**Eye Corrosion**	2298 (886/1412)	1838 (709/1129)	230 (89/141)	230 (84/142)
**Eye Irritation**	5219 (3874/1345)	4176 (3099/1077)	522 (388/134)	521 (387/134)
**Respiratory Toxicity**	1388 (835/553)	1109 (666/443)	139 (84/55)	140 (85/55)
**NR-AR**	7312 (266/7046)	5853 (213/5640)	726 (26/700)	733 (27/706)
**NR-AR-LBD**	6862 (233/6629)	5493 (186/5307)	688 (23/665)	681 (24/657)
**NR-AhR**	6603 (763/5840)	5285 (610/4675)	657 (77/580)	661 (76/585)
**NR-Aromatase**	5887 (256/5631)	4711 (205/4506)	588 (25/563)	588 (26/562)
**NR-ER**	6166 (669/5497)	4935 (536/4399)	616 (66/550)	615 (67/548)
**NR-ER-LBD**	7052 (342/6710)	5643 (274/5369)	701 (33/668)	708 (35/673)
**NR-PPAR-gamma**	6586 (197/6389)	5266 (158/5108)	661 (19/642)	659 (20/639)
**SR-ARE**	5652 (865/4787)	4521 (691/3830)	564 (87/477)	567 (87/480)
**SR-ATAD5**	7170 (249/6921)	5736 (199/5537)	718 (25/693)	716 (25/691)
**SR-HSE**	6319 (360/5959)	5059 (289/4770)	630 (35/595)	630 (36/594)
**SR-MMP**	5913 (892/5021)	4735 (713/4022)	592 (91/501)	586 (88/498)
**SR-p53**	6915 (456/6459)	5543 (364/5179)	692 (46/646)	680 (46/634)

Table S2. Scaffolds analysis of 53 datasets.

Property	Molecules / scaffolds	Ratio of scaffolds (< 5 molecules)	Property	Molecules / scaffolds	Ratio of scaffolds (< 5 molecules)
**LogS**	3.585	0.935	**hERG Blockers**	1.332	0.988
**LogD7.4**	1.832	0.948	**H-HT**	1.607	0.981
**LogP**	4.948	0.89	**DILI**	1.379	0.992
**Caco-2 Permeability**	1.604	0.972	**AMES Toxicity**	3.304	0.923
**MDCK Permeability**	1.626	0.973	**Rat Oral Acute Toxicity**	3.039	0.943
**Pgp-inhibitor**	1.572	0.977	**FDAMDD**	1.654	0.978
**pgp-substrate**	1.208	0.991	**Skin Sensitization**	2.084	0.991
**HIA**	1.443	0.989	**Carcinogencity**	1.982	0.967
**F20%**	1.391	0.989	**Eye Corrosion**	1.955	0.972
**F30%**	1.362	0.993	**Eye Irritation**	3.399	0.946
**PPB**	1.573	0.975	**Respiratory Toxicity**	1.967	0.957
**VD**	1.399	0.992	**Bioconcentration Factor**	3.343	0.925
**BBB Penetration**	1.601	0.973	**IGC50**	10.476	0.878
**Fu**	1.494	0.983	**LC50FM**	3.585	0.941
**CYP1A2 inhibitor**	1.539	0.977	**LC50DM**	2.579	0.974
**CYP1A2 substrate**	1.298	0.995	**NR-AR**	2.414	0.962
**CYP2C19 inhibitor**	1.567	0.976	**NR-AR-LBD**	2.461	0.958
**CYP2C19 substrate**	1.319	0.993	**NR-AhR**	2.36	0.962
**CYP2C9 inhibitor**	1.565	0.976	**NR-Aromatase**	2.397	0.964
**CYP2C9 substrate**	1.488	0.981	**NR-ER**	2.41	0.959
**CYP2D6 inhibitor**	1.563	0.977	**NR-ER-LBD**	2.427	0.957
**CYP2D6 substrate**	1.508	0.987	**NR-PPAR-gamma**	2.466	0.959
**CYP3A4 inhibitor**	1.548	0.977	**SR-ARE**	2.535	0.957
**CYP3A4 substrate**	1.355	0.993	**SR-ATAD5**	2.409	0.96
**CL**	1.346	0.992	**SR-HSE**	2.46	0.96
**T1/2**	1.335	0.993	**SR-MMP**	2.408	0.96
			**SR-p53**	2.443	0.96

we implemented Murcko scaffold analysis to assess the chemical diversity of compound collections. The Murcko scaffolds were extracted from the molecules by removing all substituent groups but remaining linkers and ring systems. We calculated the ratio of molecules to scaffolds and the proportion of scaffolds with less than 5 molecules to represent the chemical diversity of datasets. For most datasets, the average molecules per scaffold ranged from 1.3 to 2.5, and more than 95% of the scaffolds contained no more than five molecules. The above scaffold analysis indicated a high level of the structural diversity of the training sets, and the models developed with such datasets may have good prediction coverage for structurally diverse compounds. We also extracted the most frequent 150 scaffolds from each of the datasets to generate the related cloud grams, in which the frequency of molecules containing a particular scaffold was indicated by the size of the respective structural image. The users could see the most frequent scaffolds by clicking the corresponding View Scaffolds button (https://admetmesh.scbdd.com/resources/DA).

Table S3. Performance of classification models in training and validation sets

Property	Validation set					Training set				
	AUC	ACC	SP	Sen	MCC	AUC	ACC	SP	Sen	MCC
**Pgp-inhibitor**	0.912	0.836	0.769	0.882	0.657	1.000	0.994	0.993	0.994	0.987
**Pgp-substrate**	0.901	0.840	0.853	0.828	0.680	1.000	1.000	1.000	1.000	1.000
**HIA**	0.944	0.949	0.867	0.961	0.785	1.000	0.988	1.000	0.987	0.950
**$F_{20\%}$**	0.905	0.842	0.760	0.868	0.599	1.000	0.995	1.000	0.993	0.987
**$F_{30\%}$**	0.797	0.800	0.727	0.836	0.555	1.000	0.998	1.000	0.996	0.994
**BBB Penetration**	0.920	0.852	0.810	0.885	0.698	0.992	0.957	0.948	0.964	0.912
**CYP1A2 inhibitor**	0.948	0.886	0.876	0.896	0.771	0.972	0.914	0.898	0.932	0.828
**CYP1A2 substrate**	0.842	0.816	0.800	0.833	0.632	0.985	0.936	0.942	0.929	0.871
**CYP2C19 inhibitor**	0.925	0.854	0.825	0.889	0.712	0.952	0.877	0.845	0.916	0.758
**CYP2C19 substrate**	0.926	0.741	0.688	0.818	0.497	0.974	0.928	0.894	0.977	0.859
**CYP2C9 inhibitor**	0.905	0.820	0.792	0.876	0.635	0.960	0.880	0.849	0.942	0.755
**CYP2C9 substrate**	0.785	0.744	0.816	0.636	0.461	0.967	0.904	0.911	0.894	0.801
**CYP2D6 inhibitor**	0.882	0.809	0.816	0.780	0.515	0.973	0.884	0.866	0.958	0.715
**CYP2D6 substrate**	0.775	0.663	0.600	0.727	0.330	0.947	0.893	0.849	0.937	0.788
**CYP3A4 inhibitor**	0.921	0.842	0.824	0.869	0.683	0.960	0.891	0.869	0.922	0.781
**CYP3A4 substrate**	0.802	0.753	0.760	0.745	0.505	0.948	0.887	0.920	0.855	0.776
**$T_{1/2}$**	0.822	0.744	0.750	0.736	0.481	0.948	0.869	0.822	0.938	0.746
**hERG Blockers**	0.947	0.889	0.866	0.912	0.778	0.984	0.936	0.919	0.954	0.873
**H-HT**	0.750	0.675	0.735	0.630	0.362	0.975	0.895	0.976	0.835	0.802
**DILI**	0.849	0.708	0.583	0.833	0.430	0.998	0.981	0.984	0.979	0.963
**AMES Toxicity**	0.876	0.797	0.753	0.831	0.586	0.976	0.917	0.869	0.955	0.832
**ROA Toxicity**	0.846	0.795	0.826	0.744	0.567	0.986	0.936	0.923	0.957	0.868
**FDAMDD**	0.869	0.787	0.766	0.810	0.575	0.986	0.946	0.926	0.970	0.894
**Skin Sensitization**	0.901	0.854	0.692	0.929	0.652	0.991	0.966	0.952	0.973	0.923
**Carcinogencity**	0.694	0.619	0.566	0.673	0.240	0.974	0.909	0.876	0.942	0.817
**Eye Corrosion**	0.982	0.965	0.958	0.977	0.928	1.000	0.995	0.995	0.994	0.989
**Eye Irritation**	0.963	0.931	0.904	0.941	0.825	0.996	0.974	0.983	0.971	0.834
**Respiratory Toxicity**	0.906	0.850	0.836	0.859	0.689	0.989	0.956	0.960	0.954	0.909
**NR-AR**	0.778	0.881	0.898	0.444	0.201	0.991	0.911	0.908	0.986	0.506
**NR-AR-LBD**	0.967	0.948	0.952	0.833	0.545	0.996	0.962	0.960	0.995	0.666
**NR-AhR**	0.873	0.828	0.840	0.737	0.435	0.975	0.891	0.882	0.962	0.655
**NR-Aromatase**	0.895	0.888	0.898	0.654	0.340	0.985	0.914	0.910	0.995	0.552
**NR-ER**	0.781	0.847	0.877	0.603	0.394	0.946	0.885	0.889	0.853	0.587
**NR-ER-LBD**	0.832	0.892	0.907	0.600	0.340	0.987	0.915	0.911	0.993	0.572
**NR-PPAR-gamma**	0.957	0.884	0.887	0.800	0.345	0.989	0.918	0.916	0.994	0.495

**SR-ARE**	0.852	0.841	0.871	0.678	0.483	0.954	0.891	0.888	0.905	0.675
**SR-ATAD5**	0.882	0.913	0.923	0.640	0.348	0.991	0.936	0.934	0.995	0.573
**SR-HSE**	0.855	0.885	0.898	0.667	0.384	0.985	0.908	0.903	0.990	0.582
**SR-MMP**	0.933	0.880	0.896	0.791	0.607	0.979	0.924	0.918	0.957	0.766
**SR-p53**	0.889	0.844	0.846	0.809	0.411	0.982	0.885	0.878	0.995	0.566

Table S4. Performance of regression models in training and validation sets

Properties	Validation set			Training set		
	R^2	RMSE	MAE	R^2	RMSE	MAE
**LogS**	0.871	0.814	0.555	0.967	0.399	0.287
**LogD7.4**	0.901	0.457	0.345	0.950	0.305	0.236
**LogP**	0.957	0.387	0.261	0.980	0.257	0.193
**Caco-2 Permeability**	0.786	0.296	0.203	0.943	0.152	0.117
**MDCK Permeability**	0.662	0.301	0.233	0.934	0.140	0.105
**PPB**	0.744	0.155	0.091	0.961	0.054	0.037
**VD**	0.785	0.637	0.409	0.895	0.492	0.330
**Fu**	0.778	0.354	0.258	0.861	0.268	0.197
**CL**	0.692	2.956	1.883	0.977	0.740	0.556
**Bioconcentration Factor**	0.779	0.641	0.508	0.929	0.365	0.280
**IGC_{50}**	0.860	0.356	0.270	0.920	0.305	0.232
**$LC_{50}FM$**	0.660	0.693	0.536	0.918	0.423	0.324
**$LC_{50}DM$**	0.909	0.496	0.386	0.950	0.398	0.319

Table S5. Results of leave-cluster-out validation of classification models

Property	ACC	AUC	MCC
**Pgp-inhibitor**	0.871	0.939	0.719
**Pgp-substrate**	0.808	0.887	0.618
**HIA**	0.935	0.959	0.753
**$F_{20\%}$**	0.811	0.757	0.392
**$F_{30\%}$**	0.770	0.763	0.355
**BBB Penetration**	0.809	0.886	0.573
**CYP1A2 inhibitor**	0.900	0.96	0.800
**CYP1A2 substrate**	0.722	0.796	0.438
**CYP2C19 inhibitor**	0.869	0.920	0.693
**CYP2C19 substrate**	0.657	0.706	0.333
**CYP2C9 inhibitor**	0.875	0.908	0.608
**CYP2C9 substrate**	0.621	0.651	0.248
**CYP2D6 inhibitor**	0.843	0.907	0.561
**CYP2D6 substrate**	0.716	0.788	0.436
**CYP3A4 inhibitor**	0.862	0.936	0.704
**CYP3A4 substrate**	0.787	0.868	0.58
**$T_{1/2}$**	0.665	0.729	0.334
**hERG Blockers**	0.892	0.957	0.754
**H-HT**	0.625	0.677	0.232
**DILI**	0.767	0.877	0.556
**AMES Toxicity**	0.748	0.829	0.497
**ROA Toxicity**	0.774	0.825	0.509
**FDAMDD**	0.751	0.846	0.520
**Skin Sensitization**	0.577	0.689	0.247
**Carcinogenicity**	0.550	0.594	0.128
**Eye Corrosion**	0.881	0.960	0.759
**Eye Irritation**	0.953	0.970	0.721
**Respiratory**	0.839	0.904	0.661
**NR-AR**	0.847	0.925	0.508
**NR-AR-LBD**	0.912	0.955	0.575
**NR-AhR**	0.785	0.906	0.532
**NR-Aromatase**	0.758	0.841	0.250
**NR-ER**	0.742	0.641	0.147
**NR-ER-LBD**	0.782	0.813	0.242
**NR-PPAR-gamma**	0.898	0.897	0.283
**SR-ARE**	0.782	0.809	0.351
**SR-ATAD5**	0.844	0.836	0.196
**SR-HSE**	0.814	0.841	0.332
**SR-MMP**	0.798	0.880	0.477
**SR-p53**	0.778	0.849	0.358

Table S6. Results of leave-cluster-out validation of regression models

Property	R^2	MAE	RMSE
**LogS**	0.826	0.654	0.855
**LogD7.4**	0.873	0.409	0.537
**LogP**	0.961	0.295	0.387
**Caco-2 Permeability**	0.613	0.343	0.464
**MDCK Permeability**	0.424	0.415	0.494
**PPB**	0.769	8.577	0.134
**VD**	0.392	0.783	1.371
**Fu**	0.720	0.281	0.368
**CL**	0.301	3.034	4.437
**BCF**	0.368	0.789	1.06
**IGC_{50}**	0.743	0.402	0.549
**LC_{50}**	0.710	0.641	0.892
**$LC_{50}DM$**	0.719	0.772	1.006
